# Haptenation of Macrophage Migration Inhibitory Factor: A Potential Biomarker for Contact Hypersensitivity

**DOI:** 10.3389/ftox.2022.856614

**Published:** 2022-04-06

**Authors:** Lorena Ndreu, Samantha Sasse, Ann-Therese Karlberg, Isabella Karlsson

**Affiliations:** ^1^ Department of Environmental Science, Stockholm University, Stockholm, Sweden; ^2^ Department of Chemistry and Molecular Biology, Dermatochemistry, University of Gothenburg, Gothenburg, Sweden

**Keywords:** allergic contact dermatitis (ACD), biomarkers, contact hypersensitivity (CHS), high resolution mass spectrometry (HRMS), macrophage migration inhibitory factor (MIF), tetramethylrhodamine isothiocyanate (TRITC), *in vivo* skin exposure, murine

## Abstract

The immunological response in contact hypersensitivity is incited by small electrophilic compounds, known as haptens, that react with endogenous proteins after skin absorption. However, the identity of hapten-modified proteins seen as immunogenic remains as yet largely unknown. In a recent study, we have for the first time identified a hapten-modified protein in the local lymph nodes of mice treated topically with the model hapten tetramethylrhodamine isothiocyanate (TRITC). The TRITC modification was located on the N-terminal proline of the protein macrophage migration inhibitory factor (MIF). The focus of the current study was to investigate the presence of the same hapten-protein conjugate in blood samples from mice treated topically with TRITC. Furthermore, TRITC modifications of the two major blood proteins, namely hemoglobin (Hb) and albumin (Alb), as well as TRITC modifications of MIF other than the N-terminal proline, were examined. Following incubation with different molar ratios of TRITC, a proteomic approach was applied to characterize conjugate formation of the three aforementioned proteins, using high resolution mass spectrometry (HRMS). The targeted screening of the TRITC-treated mice blood and lymph node samples for these sites led to the identification of only the same TRITC-MIF conjugate previously detected in the lymph nodes. No Hb and Alb conjugates were detected. Quantification of both the TRITC-modified and unmodified N-terminal peptide of MIF in blood and lymph node samples gave interesting insights of MIF’s role in murine contact hypersensitivity. Incubation of MIF with four different haptens encompassing different reactivity mechanisms and potencies, showed adduct formation at different amino acid residues, suggesting that MIF can be the preferred target for a wide variety of haptens. The present study provides essential progress toward understanding of hapten-protein conjugate formation in contact hypersensitivity and identifies hapten-modified MIF as a potential biomarker for this condition. Further investigation of MIF as a target protein can be a next step to determine if MIF is a biomarker that can be used to develop better diagnostic tools and targeted therapeutics for individuals with allergic contact dermatitis.

## 1 Introduction

Contact hypersensitivity (CHS) is a T-cell mediated delayed type IV hypersensitivity reaction caused by the modification of endogenous skin proteins by small exogenous electrophilic compounds, known as haptens. The clinical manifestation of the condition, allergic contact dermatitis (ACD), includes inflammation and eczema at the site of exposure (Karlberg et al., 2008). The development of CHS is divided into the sensitization phase, taking place at first encounter with the hapten, and the elicitation phase, occurring at re-exposure to the same compound. During sensitization, the hapten covalently binds to nucleophilic amino acid side chains of skin proteins forming the hapten-protein conjugate that is recognized as immunogenic. The recognition of this conjugate as an antigen induces activation of dendritic cells (DCs) in the skin. Two subsets of DCs are present in the skin, Langerhans cells (LCs) and dermal dendritic cells (dDCs), both in their immature form before antigen recognition. Among the two, dDCs are considered the ones to possess the highest potency as antigen presenting cells (APCs) (Fukunaga et al., 2008; Peiser et al., 2012). After activation, DCs take up and ingest the hapten-modified protein while migrating *via* the lymphatic vessels to the local lymph nodes. During migration, they process the conjugate into peptide epitopes and present them on major histocompatibility complex (MHC) receptors. Once in the lymph nodes, the fragment of the hapten-protein conjugate (antigen) is presented to naïve T-cell that upon recognition begin to multiply and differentiate into antigen-specific effector and memory T-cells (Alberts, 2002). Subsequently, the antigen-specific memory and effector T-cells leave the lymph nodes and start circulating in the blood and lymph vessels. Usually, the sensitization phase includes no clinical symptoms. The elicitation phase takes place upon re-exposure to the same hapten leading to the release of cytokines and chemokines that attract the circulating effector and memory T-cells to the site of exposure in the skin (Karlberg et al., 2008; Lepoittevin, 2011; Duus et al., 2021).

Although the initiation event in CHS, i.e., the recognition of the hapten-protein conjugate as immunogenic, is well established since first introduced by Landsteiner and Jacobs in 1936 (Landsteiner, 1936), many aspects of the underlying mechanism of the condition related to this immunogenic complex remain unknown. For instance, it is still more or less unknown which skin proteins are targeted by haptens *in vivo*, what hapten-protein conjugates trigger the immune system and what is presented to the naïve T-cells during the sensitization phase. Due to the lack of such insights in the mechanism of CHS, the majority of potential biomarkers described for ACD so far, represent inflammatory conditions in general (Koppes et al., 2017). We have in a recent study identified a hapten-modified protein *in vivo*, in the local lymph nodes, after topical application of the model hapten tetramethylrhodamine isothiocyanate (TRITC) on mice (Karlsson et al., 2018). The TRITC modification was found on the N-terminal proline of the protein macrophage migration inhibitory factor (MIF) (Karlsson et al., 2018). Many haptens are very reactive chemicals and are therefore likely to modify several different proteins in the skin, a feature that probably is an important part of their ability to activate innate immune pathways. However, whether any of these haptenated skin proteins are also able to activate the adaptive immune system is difficult to know. Our rationale for studying hapten-modified proteins in lymph nodes rather than skin is that conjugates found in lymph nodes (after topical exposure) are more likely to be involved in activation of adaptive immune pathways.

MIF is one of the first cytokines identified, more than 55 years ago, in studies of the delayed type hypersensitivity reaction, as a protein secreted by T-cells which had the ability to inhibit the migration of macrophages (Bloom and Bennett, 1966; David, 1966). During the years, it has been demonstrated that MIF is not only secreted by lymphocytes, but it is expressed in most immune cells, such as B-cells, DCs, macrophages, monocytes, neutrophils, eosinophils, basophils and mast cells (Baugh and Bucala, 2002; Lue et al., 2002). MIF is expressed and stored in intracellular pools of cells and tissues that are in direct contact with the external environment of the host, such as the lung, skin and gastrointestinal tracts, as well as tissues of the endocrine system, such as hypothalamus and adrenal glands (Calandra et al., 1994; Bacher et al., 1997; Fingerle-Rowson et al., 2003). The active form of this 12.5 kDa cytokine is reported to be a homotrimer (Bacher et al., 1997). However, although the structure of MIF has been fully characterized, the knowledge about its mechanism of action is still incomplete. For instance, it is unclear if the monomer, the dimer or the trimer mediates MIF action, as suggested by different studies (Tomura et al., 1999) (Mischke et al., 1998). MIF is a pleiotropic lymphocyte, endocrine factor, as well as a macrophage cytokine with two catalytic activities, tautomerase and oxidoreductase activity (Faist et al., 1989). Its tautomerase activity requires the protein’s N-terminal proline to act as a catalytic base. Despite extensive research though, no endogenous substrate has been identified (Kudrin and Ray, 2008). Today, it is proven that MIF has a key role in both immune and inflammatory responses and it is involved in numerous conditions, such as asthma (Yamaguchi et al., 2000), rheumatoid arthritis (Leech et al., 1999), sepsis (Bozza et al., 2004), atopic dermatitis (Shimizu et al., 1997), atherosclerosis (Burger-Kentischer et al., 2002) and breast cancer (Verjans et al., 2009).

Conjugates of electrophilic compounds with proteins, i.e., protein adducts, have been extensively studied in disease monitoring as potential biomarkers. Their high abundance, as well as lack of repair mechanisms compared to DNA adducts, make them more sensitive biomarkers of exposure (Rappaport et al., 2012). The increased half-life of proteins enables accumulation of protein adducts during chronic exposure to reactive species, providing more stable measurements of exposure. Until today, adduct measurements have mainly focused in the most abundant blood proteins, human serum albumin (Alb) and hemoglobin (Hb) (Carlsson et al., 2019). In the context of CHS, a large number of mainly *in vitro* studies have been conducted aiming at identifying the most reactive sites of relevant proteins, with a wide variety of haptens. These reactive sites are also assumed to be most likely modified *in vivo*. Alb has mainly been the target in such studies, as one of the major blood proteins that is also in high abundance in the skin (Aleksic et al., 2007; Jenkinson et al., 2010; Parkinson et al., 2014; Ndreu et al., 2020; Parkinson et al., 2020a), but also Hb due to its potentials as an exposure biomarker (Ndreu et al., 2020). Jenskinson et al. (2010) demonstrated that hapten-Alb conjugates could induce T-cell proliferation in individuals sensitized to the hapten in the investigated conjugate, indicating that haptenation of Alb could be the culprit of CHS. Additionally, the presence of IgG and IgM antibodies against xenobiotics conjugated to human serum Alb in a study of serum from healthy donors, suggests that Alb conjugates can activate the immune system (Vojdani et al., 2015). The adductome of other skin proteins with haptens, such as human cytokeratin 14 and human cofilin (Aleksic et al., 2008), as well as protein lysates from keratinocyte cell lines and human skin tissue (Jacksén et al., 2010; Simonsson et al., 2011; Parkinson et al., 2018; Parkinson et al., 2020b; Bailey et al., 2021) has also been investigated. Despite the extensive mapping of the most reactive sites of relevant proteins, no protein-conjugates have been identified *in vivo* except the TRITC-MIF conjugate identified by us (Karlsson et al., 2018).

The aim of this study is to expand the research of Karlsson et al., 2018. by focusing on mapping the adductome of the two major mouse blood proteins, Alb and Hb, as well as mouse MIF, with TRITC in order to characterize the most reactive sites *in vitro*. The presence and quantity of such conjugates *in vivo*, is investigated in blood and lymph node samples from the same treated mice as the initial study of Karlsson et al., using a bottom-up proteomic approach combined with high resolution mass spectrometry (HRMS).

## 2 Materials and Methods

### 2.1 Materials

BALB/C mouse plasma in 3.8% sodium citrate and mouse red blood cells in Alsever’s solution were obtained from BioIVT (Burgess Hill, United Kingdom). Recombinant mouse MIF was purchased from R&D systems (Bio-Techne brand) (Minneapolis, MNS, United States). The N-terminal MIF peptide (PMFIVNTNVPR) and its isotopically labelled equivalent (MIF-d5) (PMF-d5-IVNTNVPR) were purchased from Peptide 2.0 (Chantilly, VA, United States). Acetonitrile (ACN), methanol (MeOH), LC-grade water and ammonium bicarbonate (AMBIC) were purchased from Thermo Fisher Scientific (Seelze, Germany). Formic acid (FA), tetramethylrhodamine isothiocyanate (TRITC) mixed isomers, tetramethylrhodamine-5- isothiocyanate (5-TRITC, G isomer), 1-chloro-2,4 dinitrobenzene (DNCB), 1,2-epoxy-3-phenoxypropane (PGE), cinnamic aldehyde (CA), 5-chloro-2-methyl-2h-isothiazolin-3-one (CMIT)/2-methyl-2h-isothiazol-3-one (MIT) mixture (3:1 ratio of CMIT and MIT), DL-dithiothreitol (DTT) and iodoacetamide (IAA) were obtained from Sigma-Aldrich (Steinheim, Germany). Sodium chloride (NaCl), potassium chloride (KCl), calcium chloride (CaCl_2_), potassium dihydrogen phosphate (KH_2_PO_4_) and sodium dihydrogen phosphate (Na_2_HPO_4_) were purchased from Merck (Darmstadt, Germany). Trypsin was obtained from Promega Corporation (Madison, WI, United States). The PierceTM BCA Protein Assay Kit was purchased from Thermo Scientific (Rockford, IL, United States). SEP-PACK Vac 1cc (50 mg) C18 cartridges were obtained from Waters Corporation (Milford, MA). 0.5 mL Amicon^®^ Ultra 3K and 10K centrifugal filters were purchased from Merck Millipore (Cork, Ireland).

### 2.2 Animals

Eight to twelve-week-old female CBA/Ca mice (B&K Scanbur, Sollentuna, Sweden) were housed in cages with HEPA-filtered airflow under standard conditions of light-, humidity- and temperature-controlled rooms. The animal experiment included the topical treatment of mice with 25 µL of TRITC (6-TRITC, R isomer) (0.25% (w/v), 5.6 mM, 12 mice or 0.50%, 11 mM, 6 mice) or vehicle (acetone:dibutyl phtalate, 1:1), on the dorsum of the ears for three consecutive days. Eighteen hours after the last exposure, the mice were sacrificed and blood as well as lymph nodes were collected and lymph node single cell suspensions were prepared. In this study, blood samples from six mice treated with TRITC and six control mice treated with vehicle were analyzed. The lymph node single cell suspension samples were from TRITC- or vehicle-treated mice pooled group-vise, 12 (0.25% (w/v), 5.6 mM) and 18 mice, respectively. The regional ethics committee, Jordbruksverket, approved all experimental protocols and the animal procedures were performed under the approved guidelines. The total protein content of each mice sample was assessed using the Pierce TM BCA Protein Assay Kit. The blood and lymph node single cell suspensions from mice were stored at −20°C before sample treatment.

### 2.3 Commercial Proteins (Alb, Hb, MIF)

Upon arrival, the red blood cells were subjected to an equivalent volume of cold Ringer’s solution (125 mM NaCl, 5 mM KCl, 1.5 mM CaCl_2_, pH = 7.4), followed by centrifugation at 500 g for 5 min at 4°C and removal of the supernatant. The washing step was repeated two times. To obtain Hb, red blood cell lysis was performed by resuspension in an equal volume of distilled water. The total protein content of the Hb fraction and the plasma was assessed using the PierceTM BCA Protein Assay Kit and measuring the absorbance at 562 nm. The amount of Alb was estimated to be 50% of the total plasma protein content. Recombinant mouse MIF protein was upon arrival, reconstituted in phosphate-buffered saline (PBS) (137 mM NaCl, 3 mM KCl, 10 mM Na_2_HPO4, 14 mM KH_2_PO_4_). Hb fraction and plasma were stored at 4°C before sample treatment. Reconstituted MIF was stored at −20°C according to the manufacturer’s instructions.

### 2.4 General Reaction Procedure of Hb, Alb and MIF With the Hapten TRITC

Hb (0.016 µmol) was incubated with TRITC at 0.1, 0.5, 1 and 5-fold TRITC to protein molar ratio. For Alb (0.015 µmol), 0.1, 0.5, 1, 5 and 10-fold molar ratio of TRITC to protein was applied. For MIF (0.32 nmol), 0.1, 1 and 5-fold molar ratio of TRITC to protein was used. All mixtures were further diluted with PBS (137 mM NaCl, 3 mM KCl, 10 mM Na_2_HPO_4_, 14 mM KH_2_PO_4_) to a final incubation volume of 100 µL. Protein controls without the addition of TRITC were also included for each protein incubation. The organic solvent of the TRITC stock solution constituted 5–5.4% of the total incubation volume. Incubations of the TRITC-protein mixtures and protein controls were performed for 24 h at 37°C. After the incubations, 100 µg protein (4 µg in the case of recombinant MIF) were added to a 3K/10K cut-off filter and diluted with 400 µL of 25 mM AMBIC pH 8.0, followed by centrifugation at 14000 g for 15 min to remove the excess of unreacted TRITC. 200 µL of 25 mM AMBIC pH 8.0 was added twice more to the cut-off filter and centrifuged under the same conditions for buffer exchange purposes. Afterwards, samples were reduced with DTT at a final concentration of 5 mM for 40 min at room temperature. After reduction, samples were alkylated with IAA at a final concentration of 7 mM for 30 min in the dark. After alkylation, trypsin (1:50 w/w) digestion was carried out for 24 h at 37°C. The digestion was stopped *via* the addition of 0.1% FA in water. The samples were further cleaned up using SEP-PACK Vac 1cc (50 mg) C18 cartridges. Initially, the SPE cartridges were conditioned with 1 mL MeOH and 1 mL ACN, followed by a washing step using three times 1 mL 0.1% FA in water. The samples were then applied on the column and washed again with 3 mL of 0.1% FA in water. The peptides were eluted with 3 times 0.4 mL 70% ACN. The eluate was evaporated to dryness and reconstituted in 100 µL 0.1% FA in water containing 5% ACN.

### 2.5 General Procedure for Topically Treated Samples From Mice

100 µg protein of each sample of whole blood and lymph node single cell suspensions from topically treated mice were diluted with 25 mM AMBIC pH 8.0 to a total volume of 100 µL. Afterwards, the samples were reduced, alkylated, digested and cleaned up under the same conditions as described in Section 2.4, with the only difference that new trypsin (1:100 w/w) was added after approximately 16 h of digestion to refresh the enzyme activity. A second subset of lymph node single cell suspensions were thermally denatured at 75°C for 30 min, prior to the protein digestion procedure.

### 2.6 Quantification of TRITC-MIF and MIF Peptides in Samples of Topically Treated Mice

Quantification of the non-adducted N-terminal MIF peptide was based on its isotopically labeled equivalent MIF-d5. 10 µL of a 100 nM solution of MIF-d5, were added to each sample before the beginning of the sample preparation, resulting in a concentration of 10 nM in the final reconstituted samples after digestion. Quantification of the adducted N-terminal MIF peptide (TRITC-MIF) was based on an external standard generated in house by incubation of standard N-terminal MIF peptide with TRITC (G5 isomer). Calibration curves were prepared in the range 0.5–4.0 nM for the MIF peptide, with the final concentration of MIF-d5 at 10 nM and 0.5–2.5 nM for the TRITC-MIF peptide. All calibration curve standards were prepared in 100 µg of digested commercial mouse serum following the exact procedure as for the samples. All samples were analyzed in triplicate for statistical purposes and the final results are expressed as pg of peptide per µg of total protein.

### 2.7 Evaluation of the Quantification Method

Limit of detection (LoD) and limit of quantification (LoQ) were determined by analysis of the analytes at low concentration, and were calculated as 3 and 10 times the signal-to-noise ratio for LoD and LoQ, respectively. The signal is the average response of three replicate analysis and noise is the standard deviation of the same replicate analysis. The linearity of the method for the two targeted peptides was assessed by the analysis of the calibration curves mentioned in the previous section. Quality control (QC) samples were based on the analysis of standard MIF peptide at two concentration levels, 0.8 and 2.3 nM, and were analyzed together with the other samples to determine the accuracy, expressed as % relative error (%RE) and precision, expressed as % relative standard deviation (%RSD) of the method. Furthermore, the losses during sample preparation were examined for TRITC-MIF, since quantification of this peptide adduct was based on an external standard. The recovery was assessed by spiking 100 µg of commercial mouse serum with a known concentration of TRITC-MIF before reduction, and three other samples during the reconstitution after digestion, and comparing the quantified concentrations. Carry-over effect was assessed by analysis of instrumental blanks along with the rest of the samples. All samples were analyzed in triplicate for statistical purposes.

### 2.8 Liquid Chromatography-High Resolution Mass Spectrometric Method (LC-HRMS)

#### 2.8.1 Liquid Chromatography Method

Analysis was performed on a Dionex UltiMate 3000 UHPLC system coupled to a QExactive Quadrupole-Orbitrap mass spectrometer from Thermo Fisher Scientific (Waltham, MA, United States). Peptides were separated on an AcclaimTM RSLC 120 C18 column (2.2 µM, 150 mm × 2.1) from Thermo Scientific (Sunnyvale, CA, United States). The mobile phase consisted of (A) 0.1% FA in Milli-Q water containing 5% ACN and (B) 0.1% FA in ACN containing 5% Milli-Q water. The column temperature was 40°C. The gradient of the LC method for the untargeted and confirmation analysis of the commercial proteins and the initial screening of the treated samples from mice was performed as follows: 0–2 min, 5% B; 2–35 min, 5–50% B; 35–35.1 min, 50–95% B; 35.1–41 min, 95% B; 41–41.1 min, 95–5% B; 41.1–45 min, 5% B. Quantification of the detected adducted peptides was performed with a shorter gradient: 0–20 min, 50% B; 20–23 min, 50–95% B; 23–27 min, 95% B; 27–27.01 min, 5% B; 27.01–30 min, 5% B.

#### 2.8.2 Untargeted Analysis of TRITC Treated Alb, Hb and MIF

Peptides were analyzed in positive ion mode using a Full MS/dd-MS^2^ experiment with an exclusion list based on the control untreated sample. Expected chromatographic peak width was set at 15 s and full MS was conducted at a resolution of 120,000, an AGC target of 1e6, a maximum IT of 30 ms and a scan range of 200–3,000 m/z. dd-MS^2^ was performed at a resolution of 30,000, an AGC target of 1e5, a maximum IT of 50 ms, an isolation window of 2 m/z and a normalized collision energy of 25. Untargeted analysis was based on one replicate for each molar ratio and control.

Raw mass spectrometry data were handled using Proteome Discoverer 2.3. In the modified PWF Basic Sequest HT processing step, the raw data were searched against the UniProtKb/Swiss-Prot mouse Alb, Hb and MIF with variable modifications of oxidation at methionine, carboxymethylation of cysteine and the added mass of TRITC (+443.1303 Da). Possible modification sites of TRITC at the amino acid residues of Cys, His, Lys, Arg, Ser, Thr, Tyr, and N-terminus of peptides were also included. In the CWF basic consensus step, the generated peptide spectral matches were grouped, validated and filtered before turning into a peptide and protein report. The validation of the peptide spectral matches was set at a strict target false discovery rate of 0.01 and a relaxed target false discovery rate of 0.05. Peptides below the high confidence level were filtered out.

#### 2.8.3 Targeted Analysis of TRITC Reacted Alb, Hb and MIF

Peptides were analyzed in positive mode using a PRM approach with an expected chromatographic peak width of 15 s. The masses of highly confident TRITC-modified peptides were included in the targeted analysis. The PRM experiment was conducted at a resolution of 30,000, an AGC target of 1e^5^, a maximum IT of 50 ms, an isolation window of 2 m/z and a normalized collision energy of 30. The targeted analysis was based on triplicate measurements for each molar ratio and control.

#### 2.8.4 Targeted Analysis of Protein Adducts in Topically Treated Samples From Mice

Peptides were analyzed in positive ion mode using a PRM experiment with an expected chromatographic peak width of 15 s. The targeted screening included the masses from the confirmed TRITC-modified Alb, Hb and MIF peptides obtained from the characterization. The PRM experiment was conducted under the same conditions as described in Section 2.8.3. The targeted screening was based on one replicate for each topically treated control and TRITC-treated sample.

The PRM method for quantification used the same setting, and included the masses for the MIF, MIF-d5 and TRITC-MIF peptides. Quantification was based on the two most intense ions for the TRITC-MIF peptide and the three most intense ions for MIF and MIF-d5, Table 2. All samples were analyzed in triplicate for statistical purposes.

### 2.9 Reactivity of MIF Toward the Haptens DNCB, PGE, CA, and CMIT/MIT

MIF protein (0.32 nmol) was incubated separately with DNCB, PGE, CA, and CMIT/MIT at 5-fold molar excess of hapten to protein. The overall reaction procedure followed was the same as described in Section 2.4. Sample analysis, both untargeted and targeted, was performed as described in Section 2.8, with the added masses of each hapten used in the untargeted analysis summarized in Table 3.

## 3 Results

### 3.1 Characterization of Alb, Hb and MIF Adductome With the Hapten TRITC

The adductome of the two most abundant blood proteins, Alb and Hb, using commercially available murine serum and erythrocytes, as well as commercial mouse MIF protein, with the model hapten TRITC was investigated. Identification of TRITC-adducted sites was performed using initially an untargeted approach, followed by confirmation with targeted analysis.

#### 3.1.1 Untargeted Proteomics Results

**FIGURE 1:**
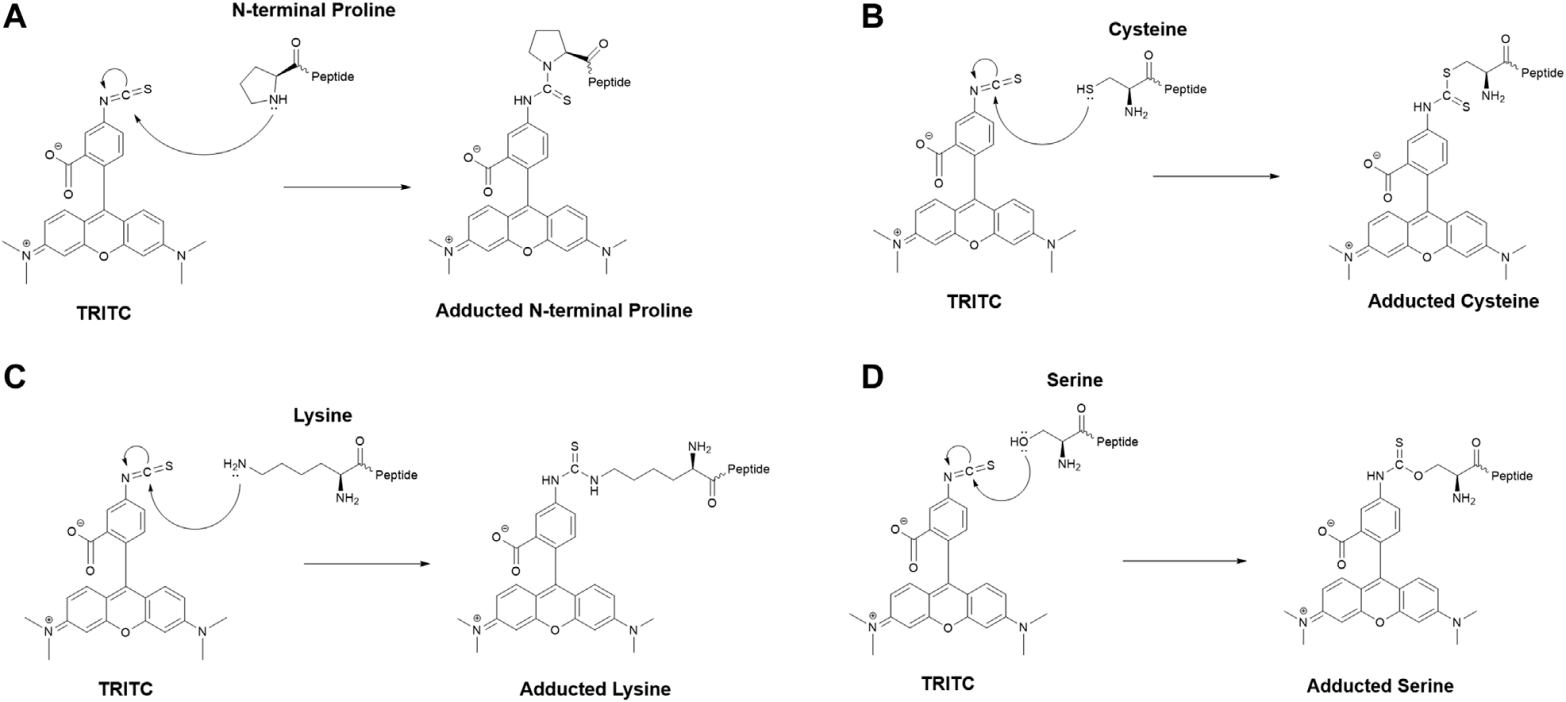
Characteristic reaction mechanism of the model hapten TRITC with different side chains of nucleophilic amino acids found modified in this study, **(A)** N-terminal proline, **(B)** cysteine, **(C)** lysine, **(D)** serine.

The MS^2^ data obtained from the untargeted analysis were processed using the added mass from the reaction with TRITC, corresponding to 443.1303 Da. The nucleophilic amino acids Cys, His, Lys, Arg, Ser, Thr, Tyr, and N-terminus were the investigated targets for all three studied proteins. Characteristic reactions of TRITC with the nucleophilic side chains of some of these amino acids are shown in Figure 1.

The untargeted results suggested two potential sites being modified by TRITC in Alb, three sites in Hb and three sites in MIF. These sites were observed to be modified at all molar ratios tested for Alb and Hb, while in MIF, only the N-terminal Pro was found to be modified at all ratios. The remaining two sites were modified at equimolar concentration of TRITC. The MS and MS^2^ spectra of the peptides containing the sites with the potential TRITC modification were initially manually evaluated. Modified peptides were confidently identified through correct observation of the charge state in the MS spectra and the b- and y- ions in the MS^2^ spectra.

#### 3.1.2 Targeted Proteomics Results

The results from the untargeted analysis were used to prepare an inclusion list with the masses of confident TRITC-modified peptides for the PRM analysis. All confirmed adducted peptides and the specific modified sites for the different proteins are shown in Table 1. The targeted analysis led to the confirmation of two adducted Lys residues in Alb, namely Lys205 and Lys218. For Hb, the targeted analysis confirmed the modification on three amino acid sites, two of them being the N-terminal Val of the α and β chain and one Ser in the β chain. Three modification sites were also confirmed for MIF, namely N-terminal Pro (Pro1), Lys77 and Cys80. The amino acid sequence of each modified peptide was confirmed through correct observation of the b- and y- ions in the MS^2^ spectra. The total ion chromatogram (TIC), as well as the MS^2^ spectra for characteristic TRITC-adducted peptides in Alb, Hb and MIF are shown in Supplementary Figures S1–S3. Since a mixture of TRITC isomers was used for these type of experiments, two peaks were observed in the TIC of all confirmed sites, an additional verification that it is a TRITC modified peptide.

**TABLE 1:** Confirmed modifications sites in the different proteins after *in vitro* incubations at different TRITC to protein molar ratios. *bold letters indicate the site of adduction.

Protein	Peptide sequence	Site of adduction	Theoretical m/z	Observed m/z	Charge state
Alb	M**K**C(+IAA)SSMQK	Lys205	721.7903	721.7891	+2
Alb	AF**K**AWAVAR	Lys218	488.2416	488.2407	+3
Hb	**V**HLTDAEK	Val1/β1 and β2	678.3086	678.3074	+2
Hb	AAV**S**C(+IAA)LWGK	Ser14/β1 and β2	717.8208	717.8175	+2
Hb	**V**LSGEDK	Val1/α	595.7635	595.7636	+2
MIF	**P**MFIVNTNVPR	Pro1	865.9127	865.9114	+2
MIF	NYS**K**LLC (+IAA)GLLSDR	Lys77	661.3148	661.3147	+3
MIF	LL**C**GLLSDR	Cys80	478.2299	478.2294	+3

**FIGURE 2:**
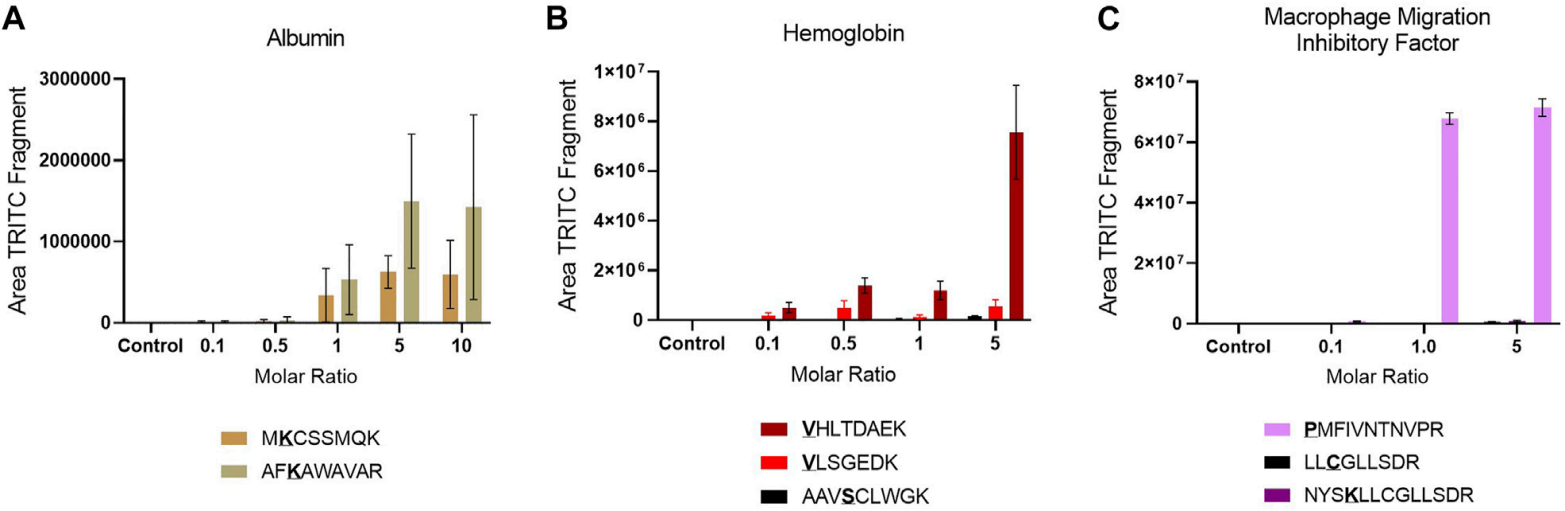
Site reactivity represented by the area of the TRITC fragment in each modified peptide in the different incubations, **(A)** Alb **(B)** Hb **(C)** MIF. Values are the mean of three replicate analysis ± STD. *Underlined bold letters indicate the site of adduction.

The reactivity of the confirmed sites was assessed by plotting the area of the characteristic TRITC fragment from the MS^2^ spectra of each adducted peptide in the different incubation with different molar ratios of TRITC used, Figure 2. An increase in area of TRITC fragment with an increase in molar ratio of TRITC, is a sign of the reactivity of the sites. Amino acid sites that show this trend and that are already modified at the lowest molar ratio of TRITC used are considered most relevant for *in vivo* screening. For Alb, the two confirmed sites were already modified at the lowest molar level of TRITC used and showed an increase in TRITC fragment with increased molar ratio of TRITC during the incubation. However, the reactivity of the two sites did not differ significantly. The standard deviation for the two modified TRITC-peptides in Alb is large compared to the modified peptides from the other two proteins, mainly due to the difference in sample complexity. For Hb, all sites were observed to be modified at all molar ratios. However, the two N-terminal Val in the *α* and *β* chain showed a higher increase in area with increased molar ratio compared to the Ser-modified peptide, with the N-terminal Val of the β chain being most reactive. For MIF, only the N-terminal Pro was modified at all molar ratios and based on the area of the TRITC fragment this was also the site that was the most reactive. Based on these results, the two TRITC-modified peptides of Alb, the two N-terminal- and the one Ser-containing peptides of Hb, and the N-terminal-containing peptide of MIF were included in the PRM list for the screening of adducts in whole blood and lymph node samples of TRITC-treated mice.

### 3.2 Targeted Screening of Modified Alb, Hb and MIF Adducted Peptides in Treated Mice Samples

After the mapping of the TRITC-Alb, TRITC-Hb and TRITC-MIF adductome and characterization of the most reactive sites *in vitro*, targeted screening was performed on samples from the topically TRITC-treated mice. The targeted analysis included TRITC-modified peptide masses containing the most reactive sites, as confirmed in Section 3.1.2. Samples from two separate animal experiments were available: whole blood from six treated mice and six controls (treated with vehicle), as well as lymph node single cell suspensions of treated mice and their equivalent control pooled group-vise.

In the whole blood sample, no Alb and Hb adducts could be detected. In contrast, the TRITC-MIF peptide could be clearly observed. The detected adduct was confirmed by comparing the retention time of the adducted peptide to the equivalent peptide from the *in vitro* incubations, Supplementary Figure S4, as well as their MS fragmentation. Two main, specific fragments that identify the TRITC-MIF conjugate are formed during the peptide fragmentation and are shown in the MS^2^ spectra. These fragments are the singly charged TRITC with a mass of 444.1386 m/z and the singly charged MIF peptide with a mass of 1,287.6873 m/z. Additionally, the TRITC-MIF conjugate was not observed in the control samples, which is an additional verification that this adduct resulted from the topical application of the hapten TRITC. To the best of our knowledge, this is the first time a hapten-protein conjugate has been identified in blood from mice after topical application of a hapten in the context of murine CHS.

No Alb or Hb adducts with TRITC could be detected in the local lymph node single cell suspension samples. However, we were able to redetect the TRITC-MIF conjugate first observed by Karlsson et al., 2018. The adducted MIF peptide was confirmed by the retention time, as well as the MS^2^ spectra that show the TRITC fragment and the MIF fragment including the relevant isotopic patterns.

### 3.3 Quantification of N-Terminal MIF and N-Terminal TRITC-MIF Peptides

Based on the positive identification of the N-terminal TRITC-MIF peptide in whole blood and lymph node samples during the targeted screening, a quantification approach was developed to quantify both the control (non-modified N-terminal MIF peptide) and the TRITC-modified N-terminal MIF peptide.

**FIGURE 3:**
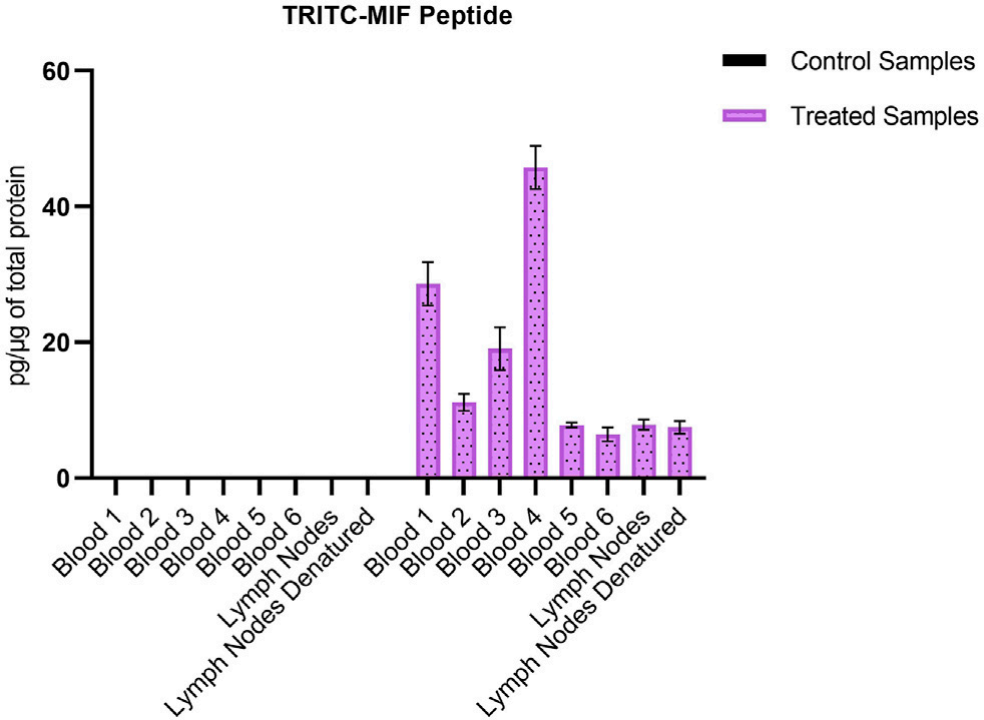
Amount of TRITC-modified MIF peptide per µg of total protein in the different whole blood and lymph node samples. Values are the mean of three replicate analysis ± STD.

Due to the lack of commercial isotopically labeled TRITC-MIF standard, as well as suitable structural analogue internal standard (IS), a TRITC-MIF conjugate was generated in house and a quantification based on external standard was implemented. The external standard, which was a purchased MIF peptide incubated with TRITC (G5 isomer) to form the TRITC-MIF peptide, was used to prepare an external calibration curve in the range of 0.5–2.5 nM, Supplementary Figure S5A. Quantification was based on the sum of the areas of the two characteristic fragments for this specific peptide, the singly charged TRITC with a mass of 444.1386 m/z and the singly charged MIF peptide with a mass of 1,287.6873 m/z. As no IS was added to the samples from the beginning to compensate for variabilities introduced during the sample preparation, a recovery experiment was performed by spiking suitable matrix before and after sample preparation, as described in Section 2.7. A recovery of 22.85% was found and this value was used for the calculation of the final concentration of TRITC-MIF in each sample to compensate for losses during sample preparation. The final concentration is expressed as pg of TRITC-modified MIF per µg of total protein in the initial sample. As shown in Figure 3, the TRITC-MIF peptide could be observed and quantified in the whole blood samples from all the six treated mice. No such adduct could be detected in any of the whole blood samples from the control mice. Concentration levels of the TRITC-MIF peptide in the different blood samples ranged from 6.5 pg per µg of total protein in treated mice number six, to 45.8 pg per µg of total protein in treated mice number four. The levels of TRITC-MIF in the lymph node single cell suspension samples were lower than the average level in the blood samples, but did not differ significantly between the denatured and non-denatured form.

**FIGURE 4:**
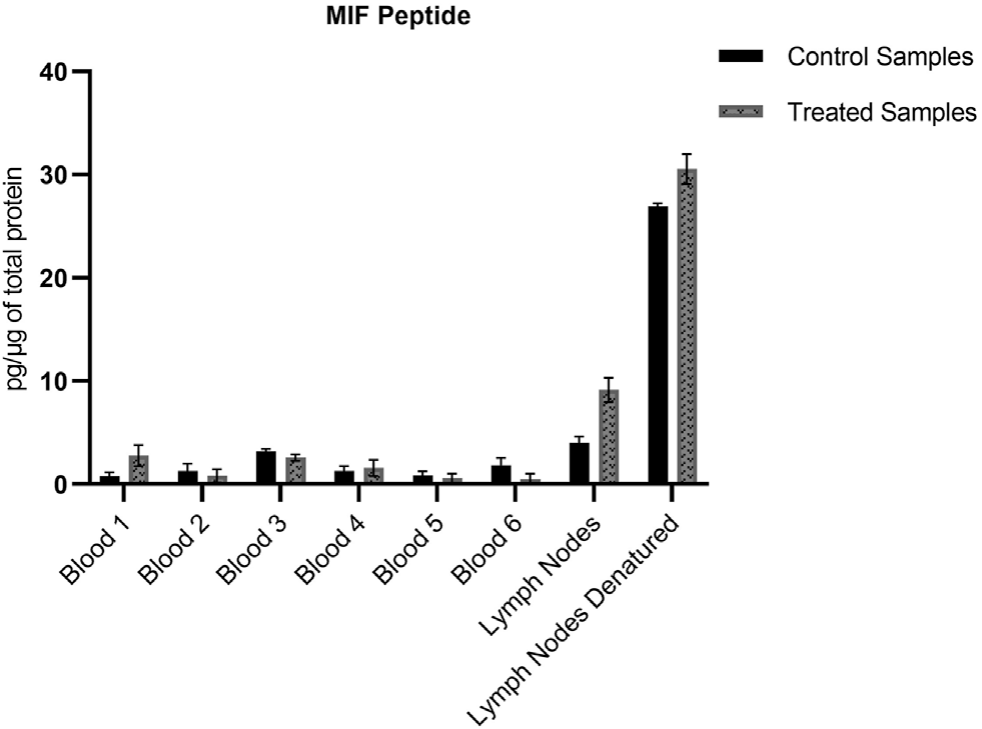
Amount of MIF peptide per µg of total protein in the different whole blood and lymph node samples. No significant difference (*p* < 0.05) could be observed for the MIF peptide between the treated and control samples. Values are the mean of three replicate analysis ± STD.

**FIGURE 5:**
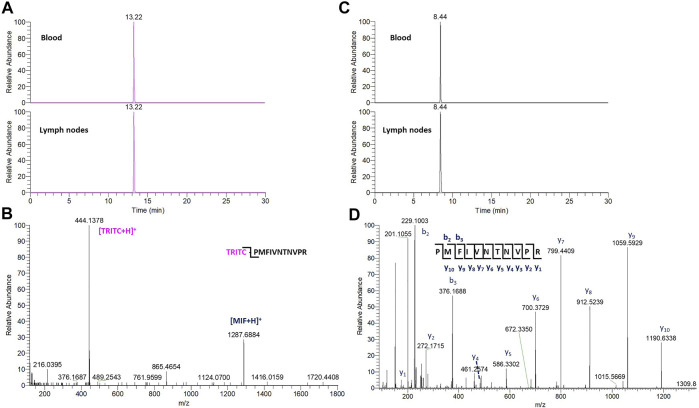
**(A)** TIC of the characteristic TRITC fragment from the TRITC-MIF peptide in blood and lymph node sample. **(B)** Representative MS^2^ spectra of the TRITC-MIF peptide. **(C)** TIC of a characteristic y ion fragment from the MIF peptide in the same blood and lymph node sample. **(D)** Representative MS^2^ spectra of the MIF peptide.

The non-modified N-terminal MIF peptide was quantified using an isotopically labelled IS (MIF-d5) that was added to each sample at a fixed concentration prior to sample preparation. Quantification was based on the sum of the areas of the three most abundant and characteristic fragments of MIF and MIF-d5, Table 2, and the response was based on the ratio of MIF/MIF-d5. The MIF peptide could be detected and quantified in all samples from TRITC-treated and control mice, Figure 4. In the whole blood samples, the levels of the MIF peptide ranged from 0.1 pg per µg of total protein in treated mice number six to 3.2 pg per µg of total protein in control mice number three. Higher concentration levels of this peptide were observed in the lymph node samples compared to the whole blood samples, especially after denaturation. This finding is associated with how the MIF protein is present in the lymph nodes and it is a clear indication of the predominance of non-free MIF in these samples. However, it is worth noticing, that no significant difference (*p* < 0.05) could be observed for the unmodified MIF peptide between the samples from TRITC-treated and control mice (Figure 4). Characteristic total ion chromatograms (TIC), as well as MS2 spectra for both MIF and TRITC-MIF peptides are shown in Figure 5.

**TABLE 2:** Specific parameters used in the quantification method for each specific compound, including exact mass of the parent ion, collision energy, fragments used for quantification.

Compound	Precursor ion [M+2H]^2+^	Product ions [M + H]^+^	Collision energy
PMFIVNTNVPR	644.3476	229.1003 (b2)	30
		799.4409 (y7)	
		1,059.5929 (y9)	
PMFIVNTNVPR-d5	646.8671	229.1003 (b2)	30
		799.4409 (y7)	
		1,064.6266 (y9)	
TRITC- PMFIVNTNVPR	865.9133	444.1378	30
		1,287.6884	

### 3.4 Method Evaluation

No peaks belonging to any of the targeted peptides were detected in any of the instrumental blanks, indicating no carry-over effect during quantification. The linearity for each peptide was assessed from the coefficient of correlation (R2), as observed in Supplementary Figures S5A, S5B, which showed a good linearity for both peptides with an R2 value of 0.9941 for the MIF/MIF-d5 peptide and 0.9782 for the TRITC-MIF peptide. Satisfactory LoD and LoQ for both targeted peptides were obtained, as shown in Supplementary Table S1. LoD was 0.1 and 0.2 nM for the unmodified N-terminal MIF peptide and TRITC-MIF peptide respectively, while LoQ was 0.5 nM for both peptides. The first point of the calibration curve for each analyte was based on their LoQ. Accuracy, expressed as %RE, and precision, expressed as %RSD, of the method, were calculated based on the analysis of the QC samples at two different levels. For the lowest level %RE was calculated to be 21.3 and %RSD 16.8, while for the higher-level QC sample %RE was calculated to be 10.4 and %RSD 11.4. In general, higher deviations in the lower level QC samples, especially during instrumental analysis that expands over several days, is considered acceptable.

### 3.5 Reactivity of MIF Toward the Haptens DNCB, PGE, CA, and CMIT/MIT

The MS^2^ data obtained from the untargeted analysis of the MIF samples incubated with the different haptens, were processed using the added masses from the reaction with each hapten, as shown in Table 3. The reaction mechanism for each individual hapten with a nucleophile showing the nature of the adduct formed in each case are shown in Supplementary Figure S6. The nucleophilic amino acids Cys, His, Lys, Arg, Ser, Thr, Tyr, and N-terminus were the investigated targets. The untargeted results suggested two sites of MIF being modified by DNCB, three by PGE, nine sites by CA (added mass corresponding to Schiff base formation), and two sites by CMIT (data not shown). The MS and MS^2^ spectra of the peptides containing the sites with the potential modifications were initially manually evaluated. Modified peptides were confidently identified through correct observation of the charge state in the MS spectra and the b- and y- ions in the MS^2^ spectra.

**TABLE 3:** Name and structure of hapten, reaction mechanism, and expected Δ Mass in Da following haptenation.

Compound name	Structure	Reaction mechanism	Δ Mass (Da)
1-Chloro-2,4-dinitrobenzene (DNCB)	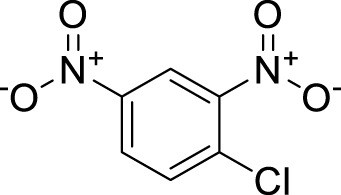	Nucleophilic aromatic substitution (S_N_Ar)	166.0015
1,2-Epoxy-3-phenoxypropane (PGE)	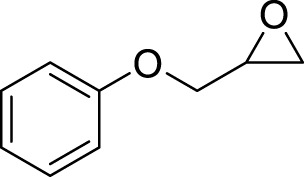	Second order nucleophilic substitution (S_N_2)	150.0681
Cinnamic Aldehyde (CA)	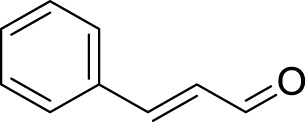	Michael addition/Schiff’s base reaction	132.0575/114.0469
5-chloro-2-methyl-2h-isothiazolin-3-one (CMIT)/2-methyl-2h-isothiazol-3-one (MIT)	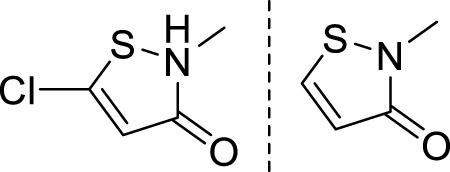	Michael addition/S_N_2 reaction at the S-atom	115.0092/112.9935/99.03203

The results from the untargeted analysis were used to prepare an inclusion list with the masses of confident MIF peptides modified by the different haptens tested for the PRM analysis. The confirmed adducted peptides and the specific modified sites for the different haptens are shown in Table 4. The PRM analysis enabled the confirmation of two MIF sites modified by DNCB, namely Pro1 and Cys80. The same sites were found to be modified by PGE, as well as one additional site, namely His88. Only Schiff bases were found to be formed from the incubation of MIF with CA, and from the nine sites suggested from the untargeted analysis, three sites were confirmed by the PRM analysis, namely Pro1, Lys77 and His88. Lastly, Lys77 and His88 were confirmed to be modified by CMIT. The amino acid sequence of each modified peptide was confirmed through correct observation of the b- and y- ions in the MS^2^ spectra. The total ion chromatogram (TIC), as well as the MS^2^ spectra for characteristic adducted peptides are shown in Supplementary Figures S7–S16.

**TABLE 4:** Confirmed modifications sites after *in vitro* incubation of MIF with the different haptens. *bold letters indicate the site of adduction.

Hapten	Peptide sequence	Site of adduction	Added Mass (Da)	Theoretical m/z	Observed m/z	Charge state
DNCB	**P**MFIVNTNVPR	Pro1	166.0015	727.3483	727.3484	+2
	LL**C**GLLSDR	Cys80	166.0015	578.2769	587.2771	+2
PGE	**P**MFIVNTNVPR	Pro1	150.0681	719.3816	719.3818	+2
	LL**C**GLLSDR	Cys80	150.0681	570.3101	570.3105	+2
	L**H**ISPDR	His88	150.0681	494.2666	494.2670	+2
CA	**P**MFIVNTNVPR	Pro1	114.0469	701.3710	701.3690	+2
	NYS**K**LLC (+IAA)GLLSDR	Lys77	114.0469	826.9,226	826.9254	+2
	L**H**ISPDR	His88	114.0469	476.2560	476.2545	+2
CMIT/MIT	NYS**K**LLC (+IAA)GLLSDR	Lys77	99.03203	819.4093	819.4200	+2
	LL**C**GLLSDR	Cys80	112.9935	551.7728	551.7735	+2

## 4 Discussion

The hapten FITC has been shown to bind to DCs in the lymph nodes after application to the skin (Macatonia et al., 1987). Due to its fluorescence, it has been used as marker for antigen binding to DC in experimental research (Weber et al., 2015). In our previous investigation (Karlsson et al., 2018), as well as the current study, we used TRITC which is an analogue to FITC with corresponding sensitizing capacity but improved physical properties, due to less overlap with autofluorescence from biological tissues. The beforementioned study, identified a TRITC-MIF conjugate in the local lymph nodes after topical treatment of mice with the potent hapten TRITC (Karlsson et al., 2018). The aim of the presented follow-up study was to examine the presence, and quantify when possible, protein adducts in blood from mice that had been topically treated with TRITC. Initially, identification of TRITC-modified sites of the most abundant blood proteins, Alb and Hb, as well as MIF, due to its relevance in our previous study, was conducted. An untargeted approach was implemented at first to identify potential adducted sites, followed by targeted analysis to confirm the adducted sites of these three proteins and assess their reactivity.

In the untargeted screening, peptides with the mass addition of TRITC, corresponding to 443.1303 m/z, were searched in the MS^2^ data of the different protein incubations. Two potential TRITC modification on Alb, (Lys205 and Lys218), three on Hb (Val1/α, Val1/β, Ser14/β) and three on MIF, (Pro1, Lys77, Cys88) were found to be potentially modified by TRITC, Table 1. Various studies that have used a similar approach, report a larger number of adducted sites for Alb and Hb with isothiocyanates (Sabbioni et al., 2001; Hettick and Siegel, 2011; Mhike et al., 2013). However, these studies used standard protein solutions that are not impacted by the matrix complexity of plasma or Hb lysate, together with haptens of lower m/z. The increased molecular size of TRITC compared to other isothiocyanates contributes to steric hindrance effects that might reduce the number of sites that can be accessed and eventually modified. All modifications observed in the untargeted screening were confirmed by targeted PRM analysis. The two N-terminals of Hb have been extensively studied and are known to be extremely reactive toward electrophilic compounds (Mhike et al., 2013; Sabbioni, 2017; Carlsson and Törnqvist, 2017). Although the hydroxyl group of Ser residues is considered a weaker nucleophile, it has been demonstrated that Ser resides of human Hb can form 4-hydroxybenzyl adducts (Rajczewski et al., 2021), while Ser residues of human Alb have been found to be modified when treated with organophosphates *in vitro* (Sabbioni and Turesky, 2017). No Lys adducts were observed for Hb, but the modified sites in Alb were exclusively lysines. Karlsson et al. (Karlsson et al., 2016) have previously shown that TRITC primarily forms stable amine adducts (Lys and N-terminals). In general, Lys residues are considered strong nucleophiles due to the amino group on the side chain. To the best of our knowledge, no previous studies have been performed on the adductome of MIF. The reactivity of the sites was assessed by the increasing area of the characteristic TRITC fragment from the MS^2^ spectra of each adducted peptide in the different incubation with different molar ratios of TRITC used, Figure 2. The most reactive sites were considered to be relevant for *in vivo* screening. For Hb, all sites were observed at the lowest molar level of TRITC and also showed increased TRITC fragment area with increased molar level of TRITC. Therefore, they were all selected for *in vivo* screening. This was also the case for the two sites confirmed to be modified in Alb. However, for MIF, the N-terminal Pro was exclusively used for *in vivo* screening, since it was the only reactive site that was already modified at the lowest molar concentration of TRITC used in the incubations.

Karlsson et al. (2018) identified for the first time a hapten-modified protein (TRITC-MIF) in the lymph node single cell suspensions from the same treated mice as used in this study. In our previous study we applied a slightly different experimental approach, in which lymph node samples were initially subjected to gel electrophoresis, and then the most intense TRITC-fluorescent band (10–15 kDa proteins) was further processed using a bottom-up proteomic approach. In the current study, the peptides containing the most reactive sites of the three proteins were used to build a PRM method in order to perform a targeted screening of tryptic digests from whole blood and lymph node samples from mice topically treated with TRITC. In both the whole blood, as well as the lymph node samples, no TRITC modified peptides of Alb or Hb could be detected. On the other hand, the TRITC-MIF conjugate previously described by Karlsson et al. could be observed in all samples from the treated mice. The peptide was identified by observation of the correct retention time compared to the *in vitro* incubations, and the two main characteristic fragments in the MS^2^ spectra, the 444.1378 m/z fragment of TRITC and the 1,287.6884 m/z fragment of the singly charged N-terminal MIF peptide. Quantification of the TRITC-MIF conjugate was possible in all blood and lymph node samples of the treated mice, but in none of the control samples, as observed in Figure 3, a clear evidence that the conjugate is formed upon skin exposure of the mice to the hapten TRITC. To the best of our knowledge, this is the first time that a hapten protein conjugate has been identified in whole blood from mice after topical application of the hapten in the context of murine CHS. Although the levels of this conjugate ranged in the blood samples from 6.5–46.5 pg per µg of total protein, no significant difference could be observed between the non-denatured and denatured lymph node samples. This is the first time such a conjugate has been identified and quantified in whole blood samples, strengthening the hypothesis that MIF conjugates play a key role in the immunological mechanism of murine CHS. The absence of TRITC-modified Alb and Hb adduct in these samples is additional evidence in favor of this hypothesis. A study by Pior et al. (1999), in which mice were painted with FITC, found that high concentrations of free FITC was seen in the blood during the first 4 h after topical exposure. Thereafter, the levels of free FITC decreased and FITC bound to proteins increased. After 6 h it was almost exclusively protein-bound FITC that was responsible for the FITC fluorescence. Thus, the study by Pior et al. indicate that isothiocyanates are able to enter the circulation after topical exposure and that they rapidly react with blood proteins. In the current study, the mice were sacrificed 18 h after the last exposure; hence, it is not surprising to find that the TRITC-fluorescence in blood is due to TRITC bound to proteins in the blood rather than free TRITC. The fact that TRITC-Alb and TRITC-Hb could not be detected in the blood from TRITC-treated mice, despite being seen in the *in vitro* incubations, could indicate that MIF was haptenated in the skin and then transported to the lymph nodes by DCs. DCs and other immune cells are known to produce high levels of MIF upon activation, but also KC are able to produce MIF (Shimizu et al., 1996) (Brocks et al., 2017). One important insight that could be useful in this context is a recently published article regarding the role of MIF in DC mobility (Ives et al., 2021). The authors demonstrated that MIF promotes migration of DCs. Leukocyte trafficking was demonstrated to occur *via* two pathways. One integrin depended, transendothelial migration, and one integrin independent, migration into lymphoid organs. In the same study, it was demonstrated that signaling involves the interaction of MIF with the CD74 receptor and activation of a Scr/PI3K pathway. It is therefore plausible that TRITC modifies MIF already in the skin. However, to prove this haptenated, MIF would have to be identified in the skin.

The unmodified MIF peptide is also of interest, to investigate the levels of MIF during an allergic response, as well as to assess the differences in protein levels between the various sample types. For the lymph node samples, an increase (approximate 3-fold in concentration) of unmodified MIF can be observed for both treated and control group, when denaturation is performed prior to sample preparation, Figure 4. Denaturing by increased temperature disrupts non-covalent interactions, such as van der Waals forces and hydrogen bonds (Bianchi et al., 2016; Zhang et al., 2016). Based on the level of increase, it can be assumed that MIF is also present in the lymph nodes in bound form. MIF is a unique protein that differs from most other cytokines in several ways. While most cytokines are usually produced upon induction, MIF is produced continuously in the cells (Bernhagen et al., 1998). MIF has a characteristic secretion pathway. It is stored in intracellular pools and is released upon stimulation. However, the exact secretion mechanism is not completely understood. It has been hypothesized that MIF must be secreted *via* a non-conventional pathway by immune and non-immune cells, since it does not carry an N-terminal leader or internal signal sequence (Bernhagen et al., 1998). Karsten et al. (2018), have demonstrated that at least in the case of MIF in RBCs, the majority of the protein was found in the cytosol, but a fraction, approximately 6% was found in the membrane fraction, suggesting that MIF can be bound both to the membrane and present intracellularly. However, in the aforementioned study, no clear conclusion could be formed if MIF resides on the membrane of RBCs or if it is bound to a receptor. MIF’s concentration in murine plasma ranges between 5 and 10 ng/mL (Li et al., 2020) with the total murine plasma protein content being 80–90 μg/μl (Ritorto and Borlak, 2008). In humans, MIF plasma concentration in healthy individuals is 24.7 ± 13.2 ng/mL (Sobierajski et al., 2013) with the average plasma protein concentration being 60–80 mg/mL (Leeman et al., 2018). Although MIF levels are usually monitored in plasma, there is more and more evidence in literature that MIF is present in greater amount in RBCs than in plasma. Sobierajski et al. (2013) were the first ones to quantify these levels and demonstrated that the concentration of MIF in RBCs is 25 μg/mL, i.e., 1000-fold higher than in plasma. MIF levels have been found to be elevated in several conditions, including rheumatoid arthritis (RA), cancer, atherosclerosis and diabetes (Lue et al., 2002). However, all these studies refer to total MIF and do not discriminate between modified and non-modified MIF. We also found, as observed in Figure 7, that when total N-terminal MIF peptide levels, both modified and unmodified, are plotted together, it is clear that the total MIF levels increased during the allergic response in the treated mice. Interestingly, we found that unmodified MIF levels are not significantly different between control and treated mice. The elevated levels of MIF during the allergic response are thus solely caused by hapten-modified MIF.

**FIGURE 6:**
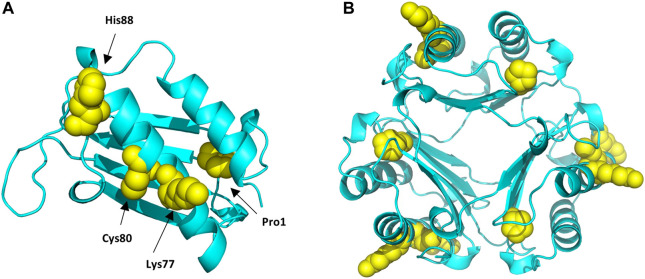
Protein Data Bank (PBD) structure of the MIF monomer **(A)**, and trimer **(B)** structure with the different sites modified by the different haptens tested highlighted in yellow.

**FIGURE 7:**
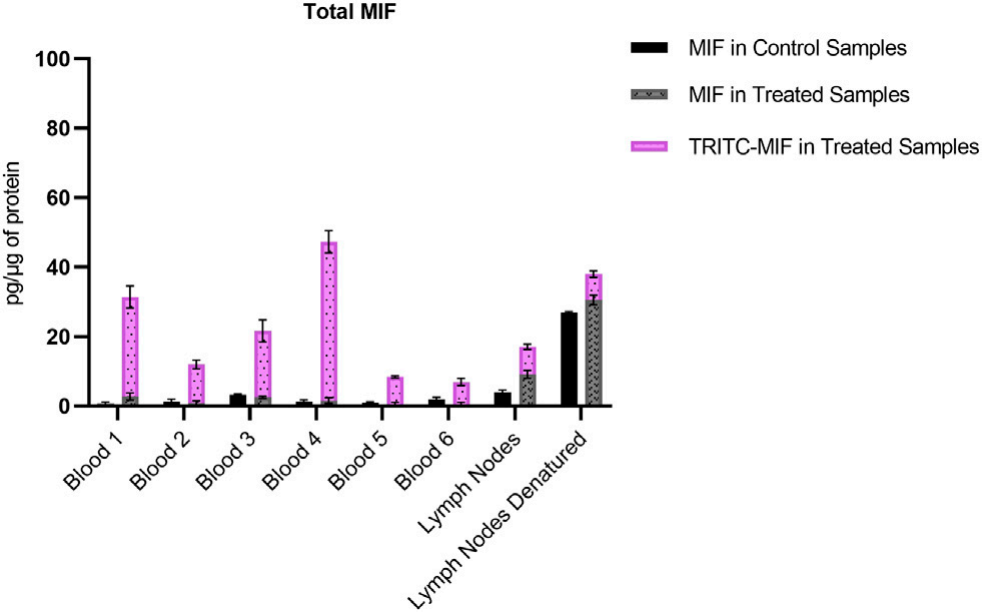
Comparison of the total amount of MIF, both as adducted and non-adducted MIF, in the control and treated samples analyzed. Levels expressed as pg per µg of total protein.

Haptens display a wide diversity considering their chemical nature, potency, as well as preferential reactivity mechanism toward different nucleophilic sites of proteins. As it is crucial that potential biomarkers described for CHS can facilitate haptenation by a large group of haptens, adduct formation of MIF with four additional haptens was investigated *in vitro*. From the haptens tested, DNCB is classified by the Local Lymph Node Assay (LLNA) as an extreme sensitizer and reacts *via* an aromatic nucleophilic substitution mechanism (S_N_Ar) (Basketter et al., 2000). PGE, a model for diglycidyl ether of bisphenol A (DGEBA), is classified as a strong sensitizer that reacts *via* a second order nucleophilic substitution mechanism (S_N_2) (Niklasson et al., 2009). CA, one of the most common contact allergens in cosmetics, is also classified as a strong sensitizer by the LLNA and since it possesses two electrophilic sites it is expected to react either *via* Schiff base formation or *via* Michael addition (Niklasson et al., 2013). In the mixture CMIT/MIT, CMIT is classified as an extreme sensitizer and MI as a moderate in the LLNA. Both are suggested to react either *via* a Michael addition or *via* an S_N_2 reaction at the S-atom (Roberts et al., 2007), giving rise to potentially different added masses, Supplementary Figure S6. Incubation of MIF with these four characteristic sensitizers, showed that all were able to modify MIF. From the nucleophilic sites of MIF, the N-terminal proline was found to be modified by DNCB, PGE and CA analogous to TRITC. Another reactive site, Cys80 was modified by DNCB, PGE and CMIT. Two more sites in the MIF sequence were confirmed to be modified, namely Lys77 and His88. Lys77 reacted with CA and CMIT, while His88 reacted with PGE and CA, Figure 6. Based on these results, it can be concluded, that MIF might be the preferred target for a wide range of haptens.

A study by Shimizu et al. (2003) elegantly showed that the CHS response to oxazolone and DNCB was impaired in MIF knock-out (KO) mice. They also found that lymph node cells from the MIF KO mice, sensitized with oxazolone and DNCB, displayed decreased capacity for transferring the CHS response compared to wild-type (WT) mice. In addition they found that MIF KO mice did not display the same significant migration of LC from the epidermis as was observed in WT mice. Although, that study does not identify any hapten-MIF conjugates, it does show that MIF is crucial for the murine CHS response for oxazolone and DNCB, haptens that belong to other mechanistic reactivity domains than TRITC. It also strengthens the hypothesis that haptenation of MIF takes place in the skin, followed by migration to the local lymph nodes by DCs (Shimizu et al., 2003). There are several studies that have shown that protein haptenation is a reversible process (Randall et al., 2013; Kumagai and Abiko, 2017; Parkinson et al., 2020b). We (Karlsson et al., 2016) as well as others (Nakamura et al., 2009), (Fleischel et al., 2009) have shown that isothiocyanates react quickly with cystine moieties, but the formed dithiocarbamates are unstable under physiological conditions and over time the dithiocarbamates degrade and stable adducts are instead formed with amine nucleophiles (thioureas). The initial cysteine binding could however be of importance as it prevents hydrolysis of the isothiocyanates and may facilitate transport of the haptens into viable epidermis, where they can form stable thiourea adducts with amine moieties. Our studies show that haptenation of MIF, at least modification of the N-terminal Pro, leads to very stable adducts (Karlsson et al., 2016; Karlsson et al., 2018). Hence, hapten-MIF adducts are stable enough under physiological conditions to go through the process of transfer from the skin to the local lymph nodes, which is often considered a requirement for activation of the adaptive immune response. Haptens ability to modify glutathione and various cysteine moieties, although reversible, could however be important for activation of innate immune pathways.

Identification of hapten-protein conjugates are of great importance, since they can lead to the development of blood-based diagnostic tools for CHS that can replace the standard patch testing, which has been the standard diagnostic method of the condition in dermatology clinics for more than 50 years. During patch testing, the most common contact allergens diluted in petroleum or water, also referred to as the baseline series, are applied for two or three days in non-sensitizing concentrations on the back of dermatitis patients with suspected ACD. Patients that are allergic to a test compound develop a delayed-type allergic reaction at the specific site where that chemical is applied (Karlberg et al., 2008; Uter et al., 2018). One of the main shortcomings of patch testing is the limitation in the number of compounds that can be applied on the patient’s back leading to high risks of missing the relevant chemical that causes the allergic response. To overcome this the baseline series has been supplemented with specific series of contact allergens based on individual exposures. However, there are still patients that are not efficiently diagnosed due to limited access of chemicals because of restrictions in licensing of manufacturers (Uter et al., 2018). Additionally, there is the adverse health effects on the patients from patch testing, since mild allergic symptoms cannot be avoided at the site of exposure.

MIF is an evolutionary well-conserved protein that has been studied extensively in murine *in vivo* models and human *in vitro* systems. It has an essential role in the pathophysiology of several allergic skin disorders including atopic dermatitis (Yoshihisa et al., 2011), zinc-allergic contact dermatitis (Yanagi et al., 2006) and pollen dermatitis in mice (Nagata et al., 2015). In addition, there are a large number of studies that have showed that elevated levels of MIF can be detected in patients suffering from various inflammatory and autoimmune conditions, such as RA (Bae and Lee, 2018) and systemic lupus erythematosus (SLE) (Foote et al., 2004). MIF-based therapeutics, ranging from small molecule inhibitors to anti-MIF antibodies, have for instance showed promise in SLE, RA and cancer (Bloom et al., 2016; Bilsborrow et al., 2019). The present study provides new insights regarding hapten-protein conjugate formation and identifies MIF as a potential mediator of CHS in mice. Considering the relevance of MIF in patients suffering from autoimmune diseases such as SLE and RA, we are of the opinion that the presence of hapten-modified MIF in patients with ACD are worth exploring to investigate if haptenated MIF could be a general disease biomarker for the condition. New diagnostic tools based on blood samples will provide easier and more efficient identification of the relevant chemical causing the allergic response in each patient.

## Data Availability

The datasets presented in this study can be found in online repositories. The names of the repository/repositories and accession number(s) can be found below: ProteomeXchange PXD031143 and PXD031793.
