# Supplementary material for: Haptenation of Macrophage Migration Inhibitory Factor: A Potential Biomarker for Contact Hypersensitivity
Source: Front Toxicol. 2022 Apr 6;4:856614. doi: 10.3389/ftox.2022.856614 (PMC9019732; doi:10.3389/ftox.2022.856614)
Supplement: Supplementary file 1 [file DataSheet1.docx]

Supplementary Material

# Supplementary Tables and Figures

**Table 1**: Limit od detection (LoD) and limit of quantification (LoQ) for the unmodified and TRITC modified MIF peptide used in the quantification method.

|  | **MIF peptide (nM)** | **TRITC-MIF peptide (nM)** |
| --- | --- | --- |
| **LoD** | 0.1 | 0.2 |
| **LoQ** | 0.5 | 0.5 |


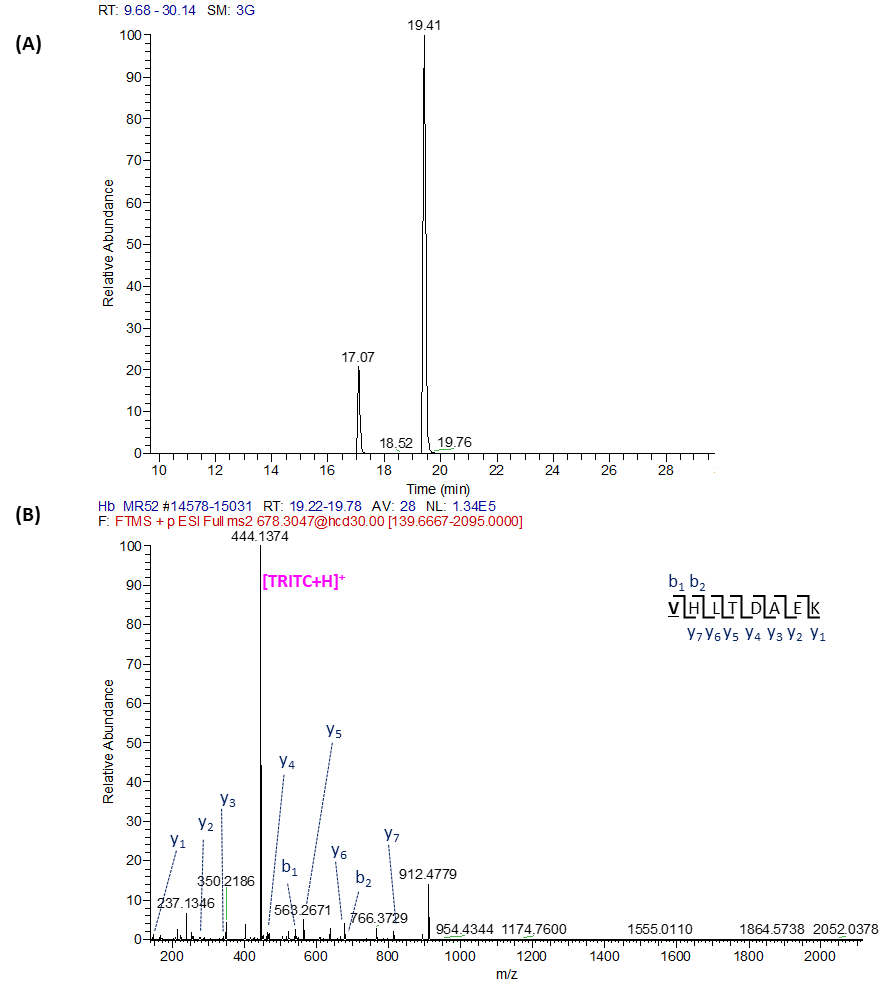


**Figure S1**: Characteristic total ion chromatogram (TIC) in (A) and tandem mass spectra in (B), of the adducted Hb peptide VHLTDAEK containing the Val1/ β1 and β2 adducted site, identified using PRM analysis. B-and y-ions are annotated in the MS^2^ spectrum. The double peak in the TIC is due to the fact that incubations were performed using standard TRITC containing both isomers. **Underlined bold letters indicate the site of adduction*


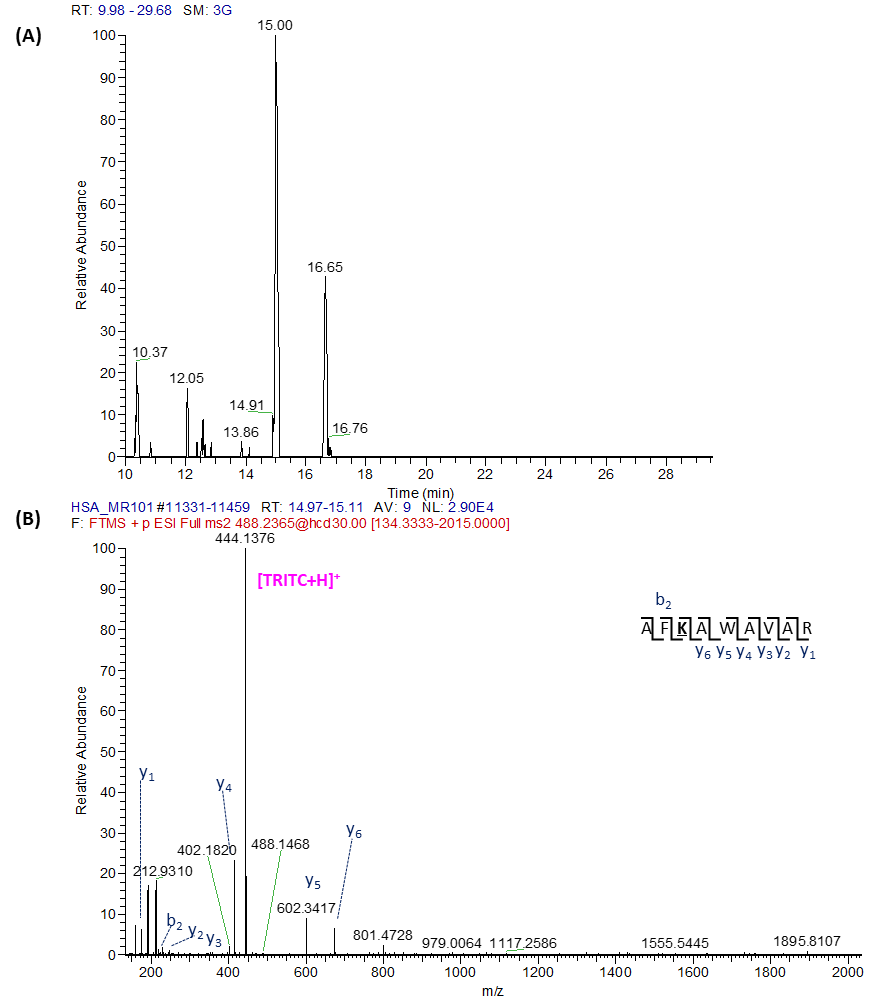


**Figure S2**: Characteristic total ion chromatogram (TIC) in (A) and tandem mass spectra in (B), of the adducted Alb peptide AFKAWAVAR containing the Lys218 adducted site, identified using PRM analysis. B-and y-ions are annotated in the MS^2^ spectrum. The double peak in the tic is due to the fact that incubations were performed using standard TRITC containing both isomers. **Underlined bold letters indicate the site of adduction*


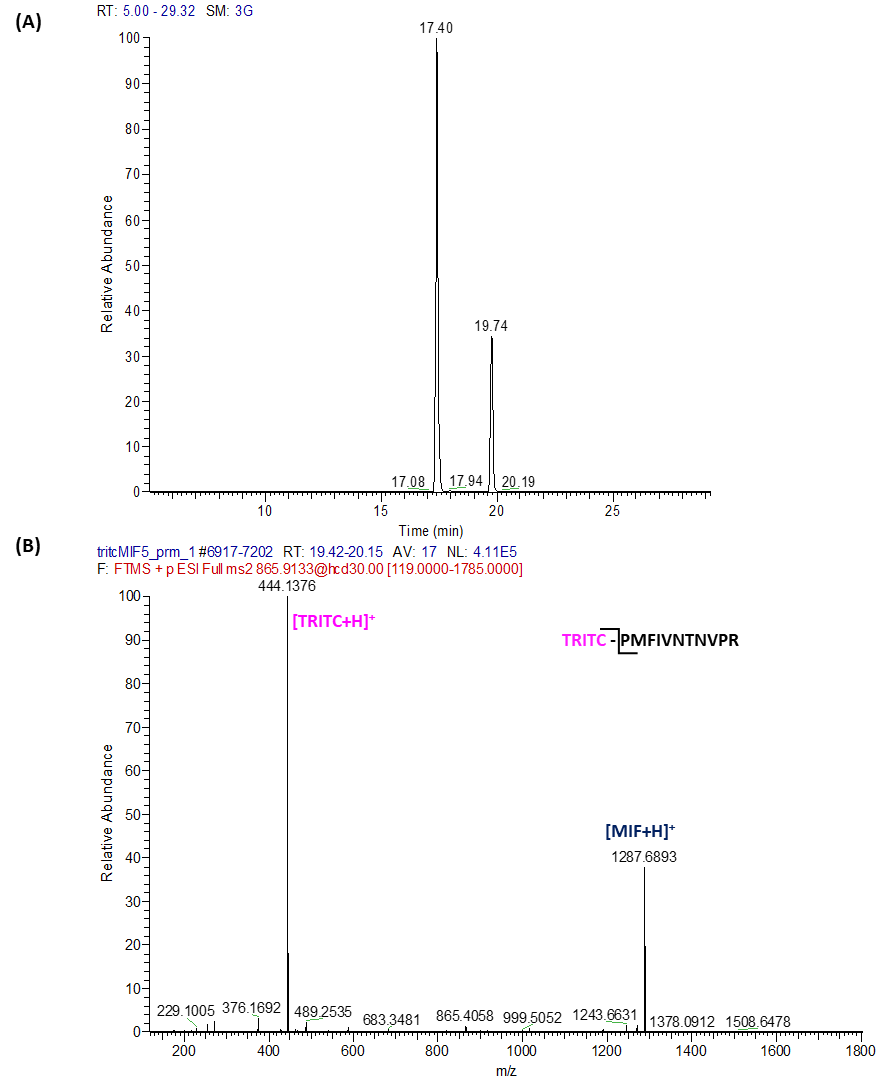


**Figure S3**: Characteristic total ion chromatogram (TIC) in (A) and tandem mass spectra in (B), of the adducted MIF peptide PMFIVNTNVPR containing the Pro1 adducted site, identified using PRM analysis. B-and y-ions are annotated in the MS^2^ spectrum. The double peak in the tic is due to the fact that incubations were performed using standard TRITC containing both isomers.


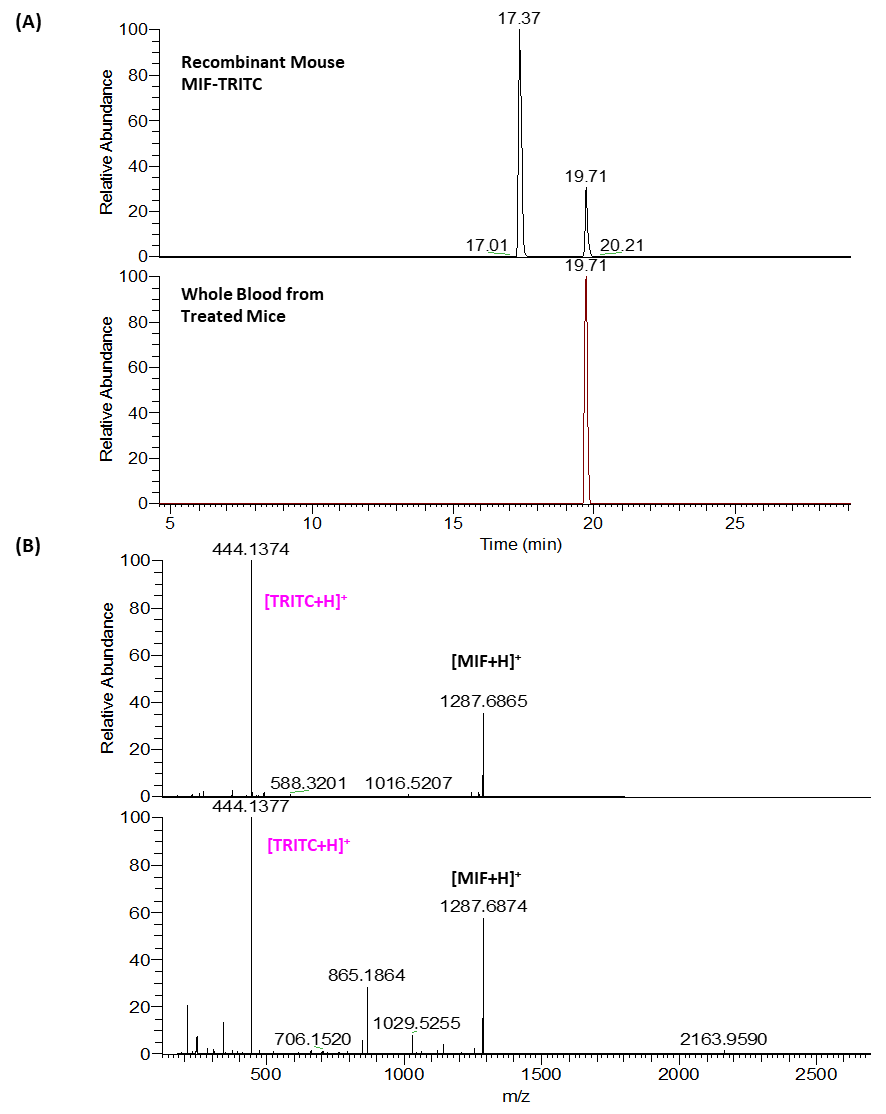


**Figure S4**: Total ion chromatograms of the recombinant TRITC MIF peptide from incubation of recombinant mouse MIF protein with TRITC *in vitro* and whole blood sample from TRITC treated mice during the targeted analysis (A) and their equivalent MS^2^ spectra. Peptides have the exact same retention time and identical fragmentation pattern. Observation of one single peak in the treated mice sample is due to the fact that mice where treated with a single TRITC isomer.

**
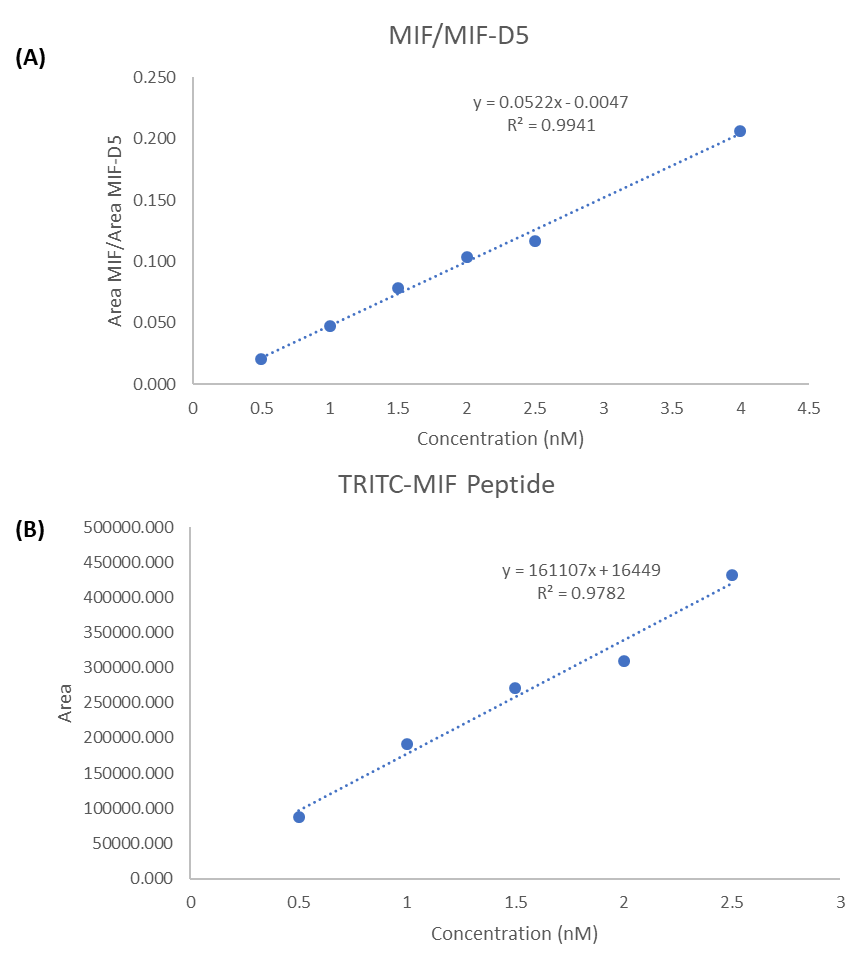
**

**Figure S5**: Calibration curves of the MIF (A) and TRITC-MIF peptide (B).


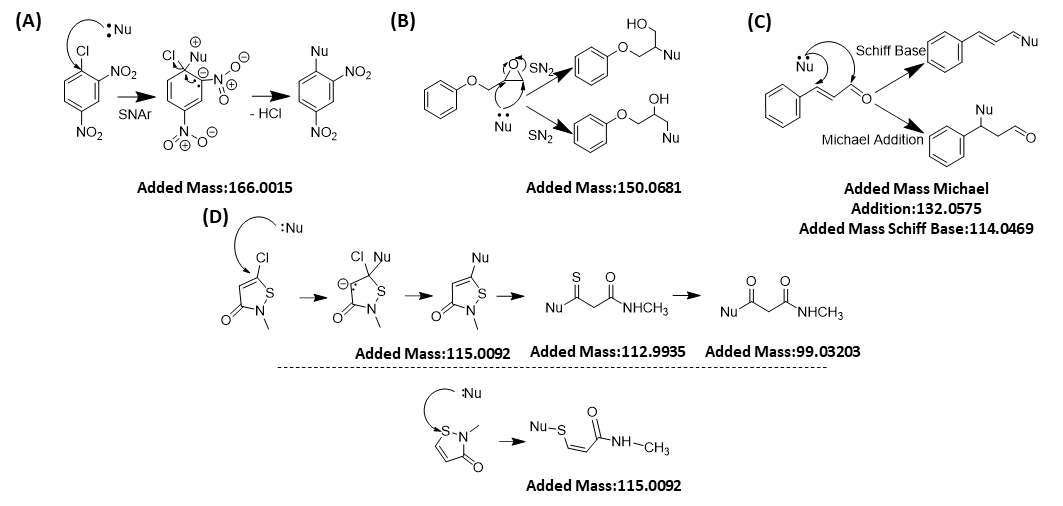


**Figure S6**: Reaction mechanisms of the different haptens incubated with recombinant mouse MIF, (A) DNCB, (B) PGE, (C) CA, (D) CMIT/MIT.


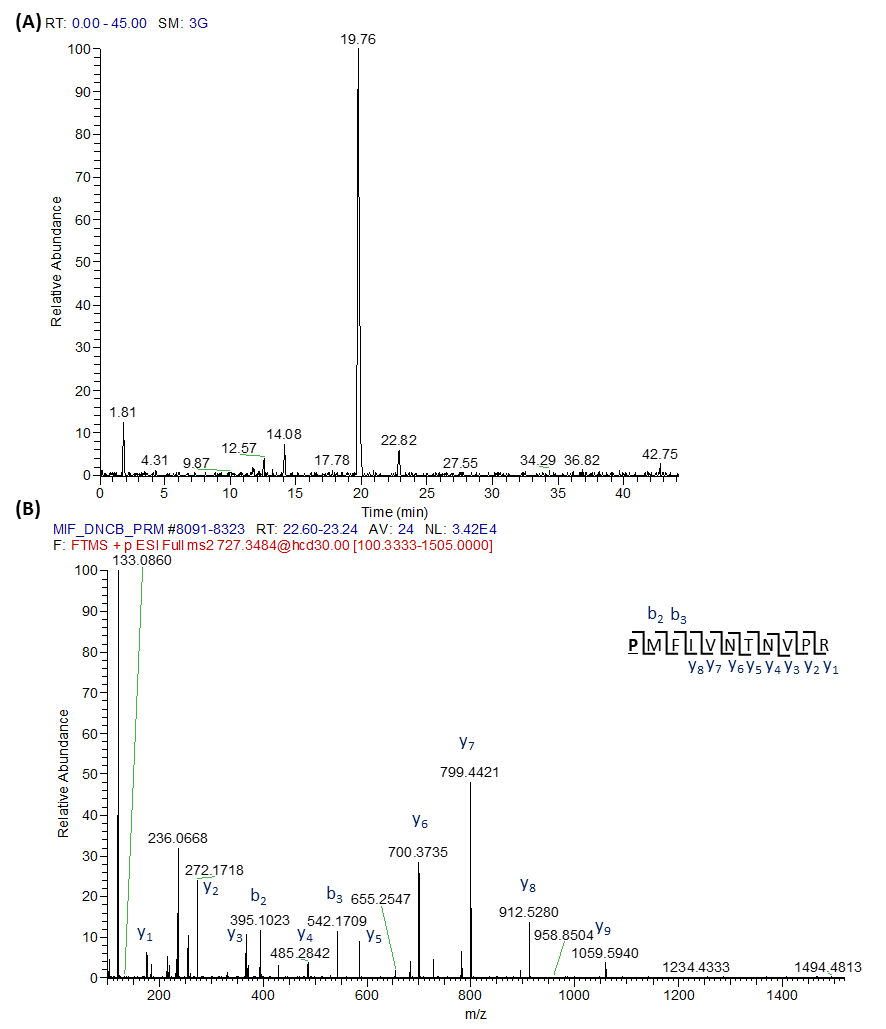


**Figure S7**: Characteristic total ion chromatogram (TIC) in (A) and tandem mass spectra in (B), of the adducted MIF peptide PMFIVNTNVPR in position Pro1 after incubation with DNCB, identified using PRM analysis. B-and y-ions are annotated in the MS^2^ spectrum.


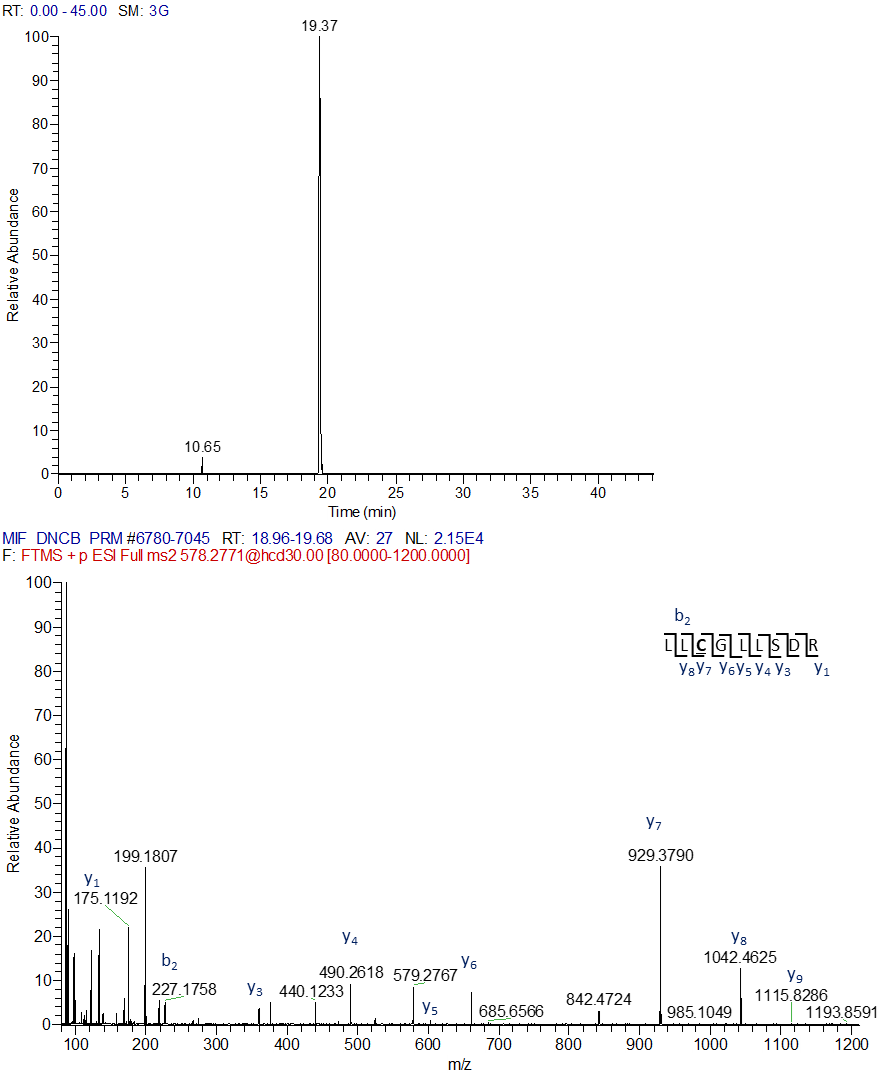


**Figure S8**: Characteristic total ion chromatogram (TIC) in (A) and tandem mass spectra in (B), of the adducted MIF peptide LLCGLLSDR in position Cys80 after incubation with DNCB, identified using PRM analysis. B-and y-ions are annotated in the MS^2^ spectrum.


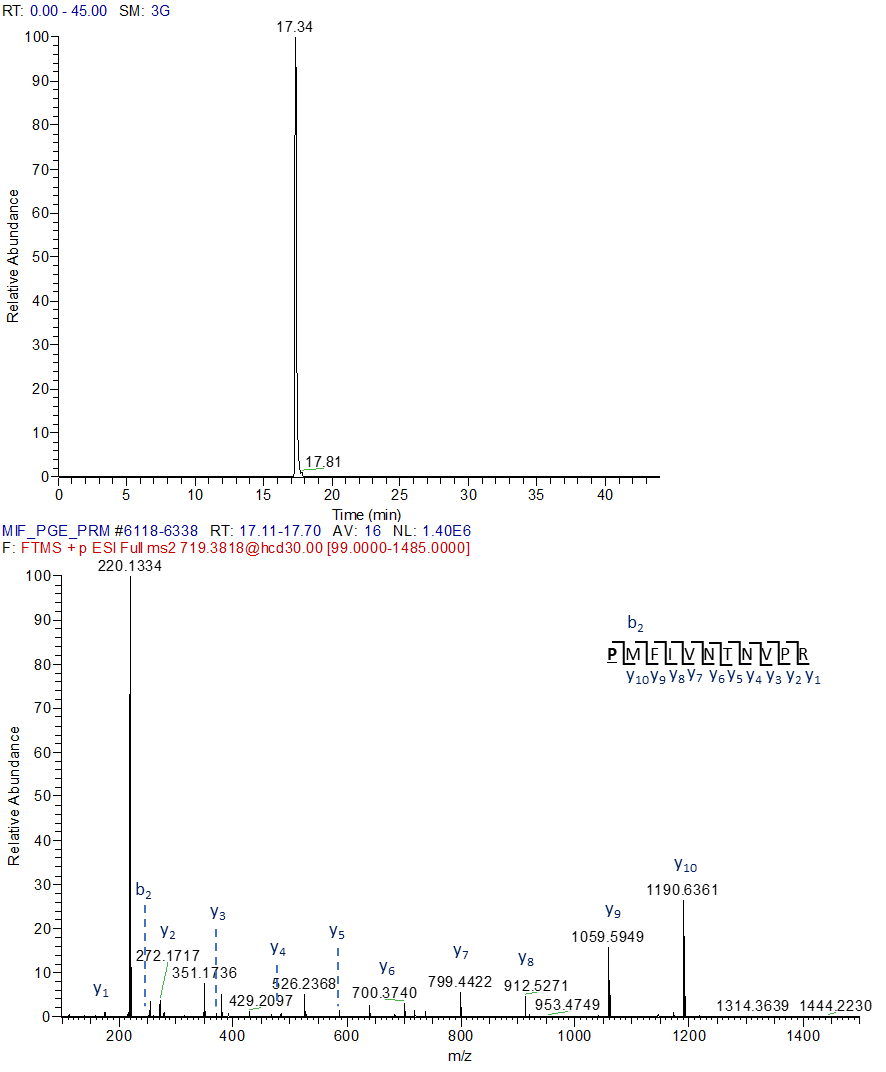


**Figure S9**: Characteristic total ion chromatogram (TIC) in (A) and tandem mass spectra in (B), of the adducted MIF peptide PMFIVNTNVPR in position Pro1 after incubation with PGE, identified using PRM analysis. B-and y-ions are annotated in the MS^2^ spectrum.


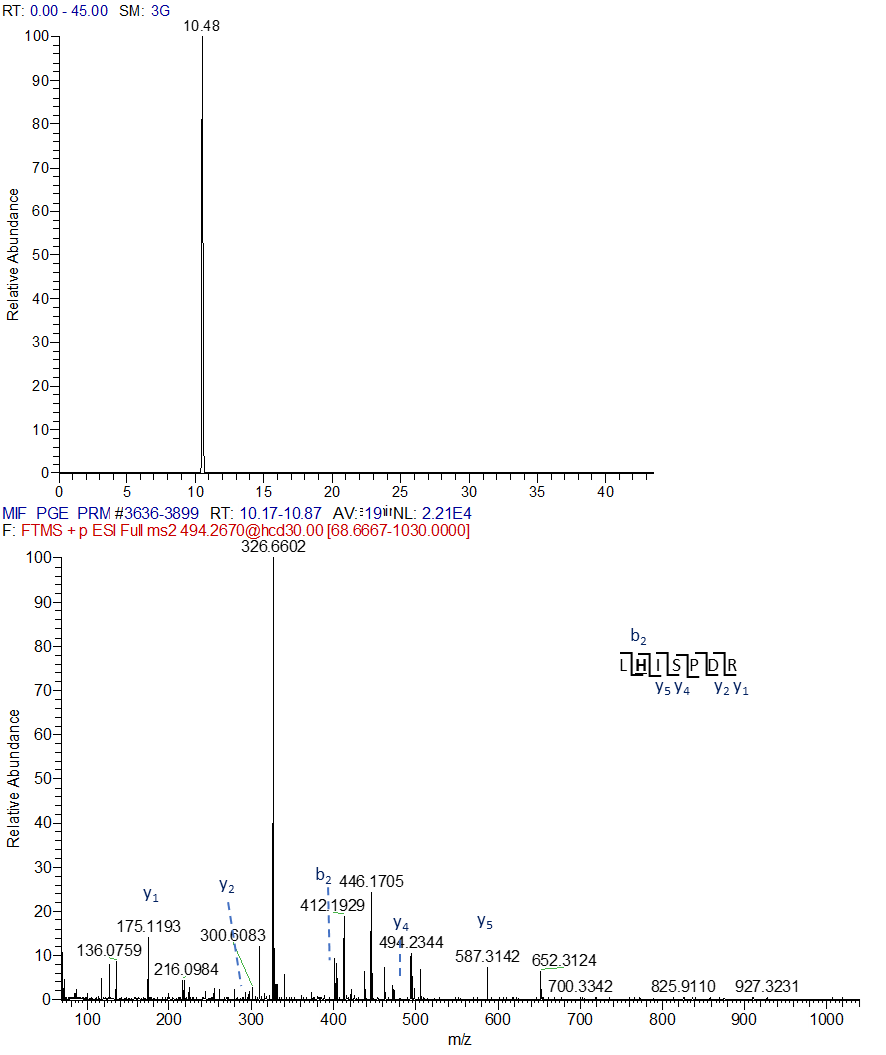


**Figure S10**: Characteristic total ion chromatogram (TIC) in (A) and tandem mass spectra in (B), of the adducted MIF peptide LHISPDR in position His88 after incubation with PGE, identified using PRM analysis. B-and y-ions are annotated in the MS^2^ spectrum.


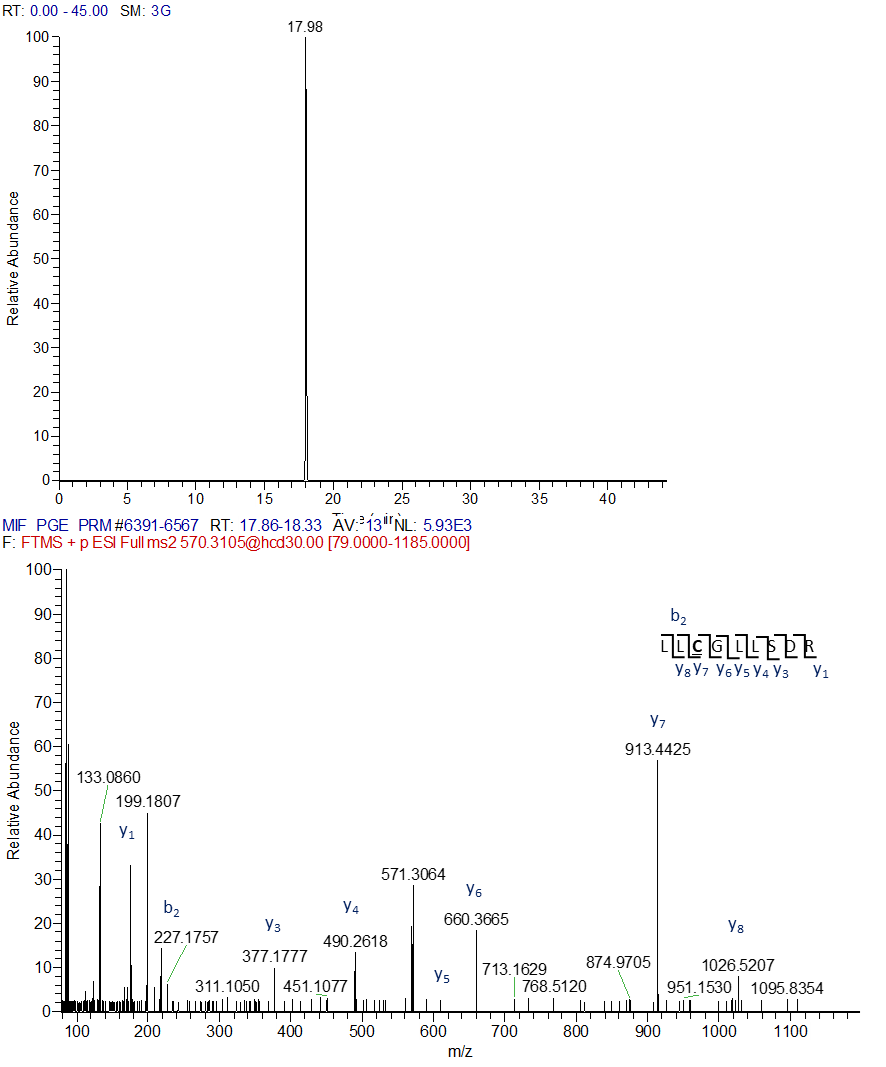


**Figure S11**: Characteristic total ion chromatogram (TIC) in (A) and tandem mass spectra in (B), of the adducted MIF peptide LLCGLLSDR in position Cys80 after incubation with PGE, identified using PRM analysis. B-and y-ions are annotated in the MS^2^ spectrum.


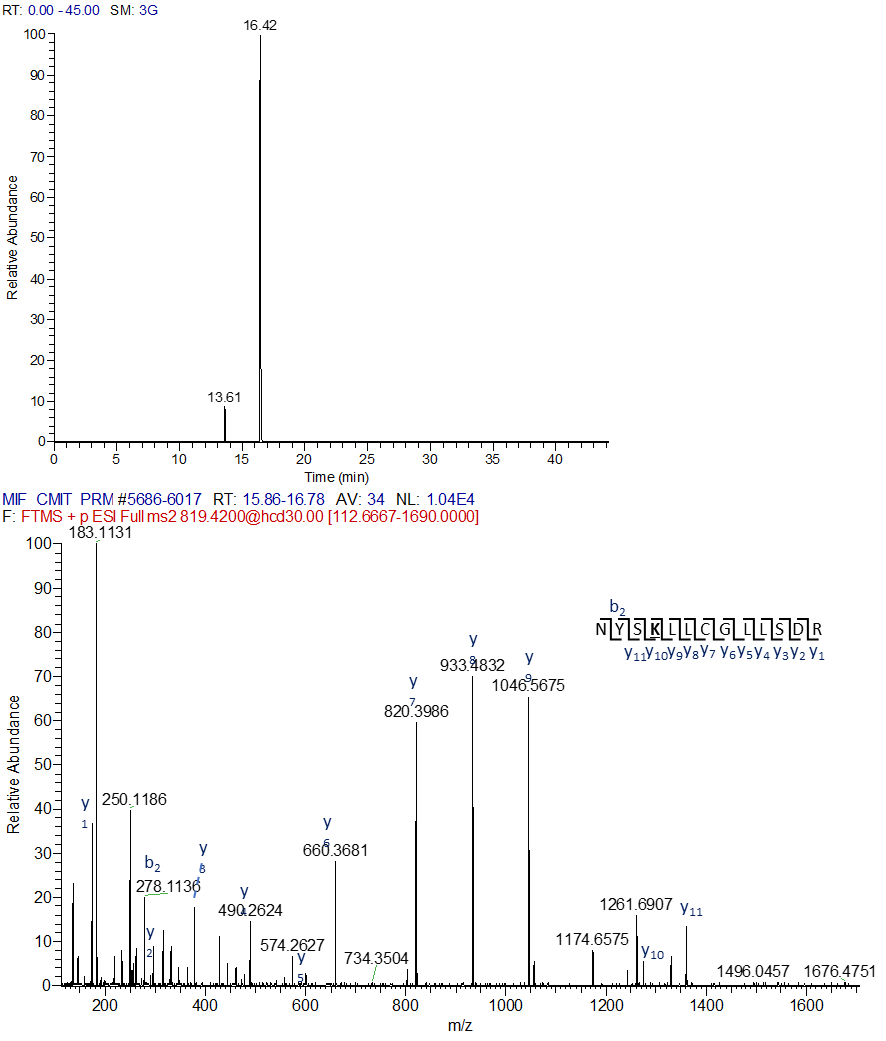


**Figure S12**: Characteristic total ion chromatogram (TIC) in (A) and tandem mass spectra in (B), of the adducted MIF peptide NYSKLLCGLLSDR in position Lys77 after incubation with CMIT/MIT, identified using PRM analysis. B-and y-ions are annotated in the MS^2^ spectrum.


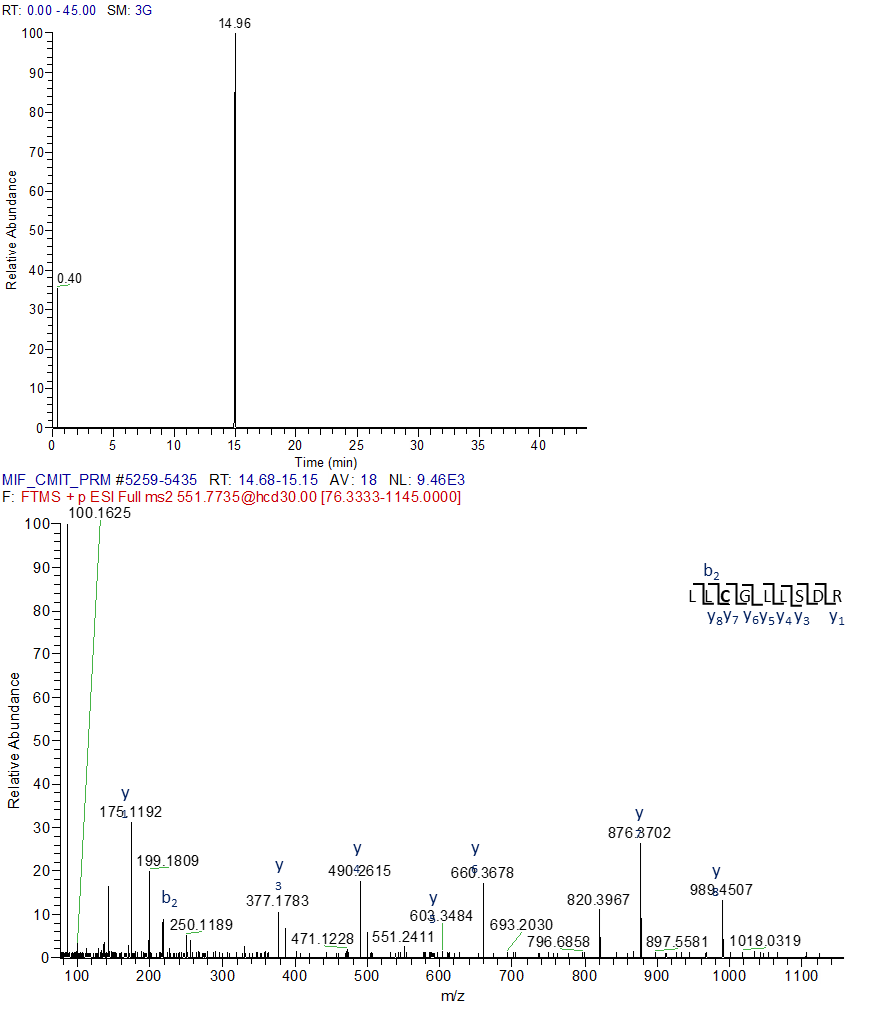


**Figure S13**: Characteristic total ion chromatogram (TIC) in (A) and tandem mass spectra in (B), of the adducted MIF peptide LLCGLLSDR in position Cys80 after incubation with CMIT/MIT, identified using PRM analysis. B-and y-ions are annotated in the MS^2^ spectrum.


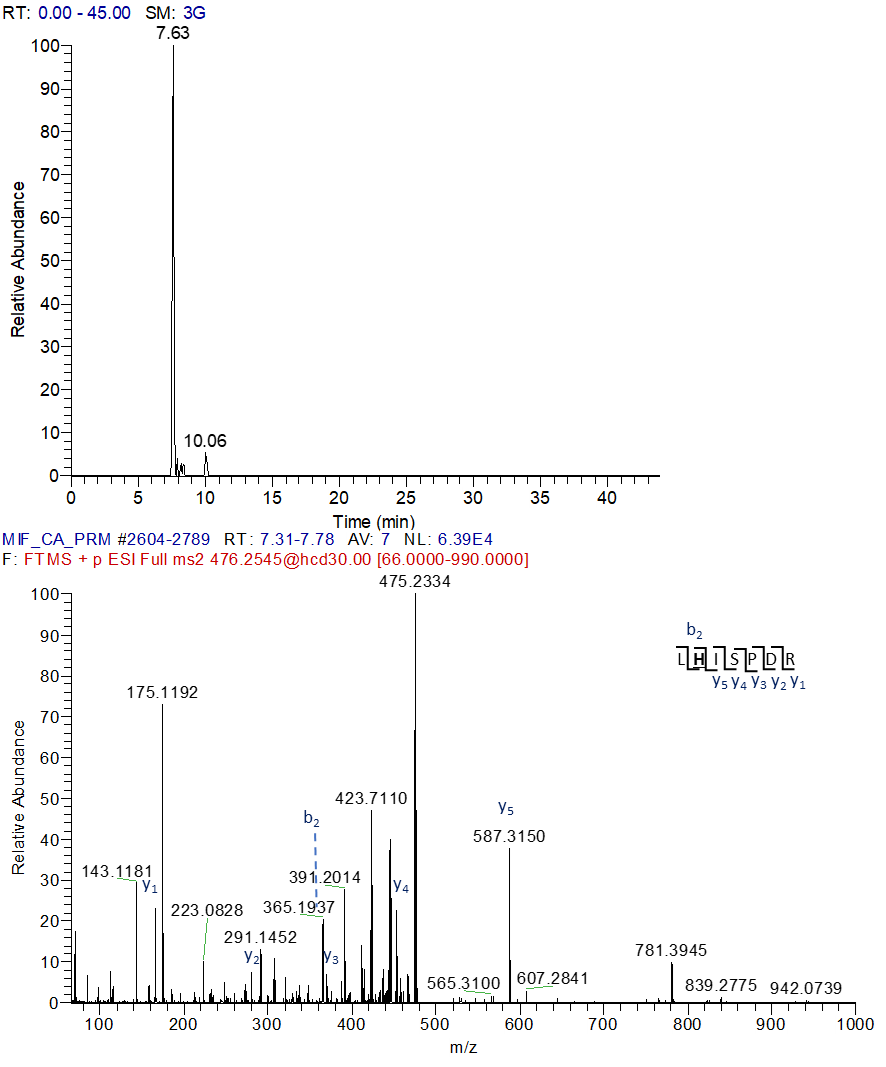


**Figure S14**: Characteristic total ion chromatogram (TIC) in (A) and tandem mass spectra in (B), of the adducted MIF peptide LHISPDR in position His88 after incubation with CA, identified using PRM analysis. B-and y-ions are annotated in the MS^2^ spectrum.


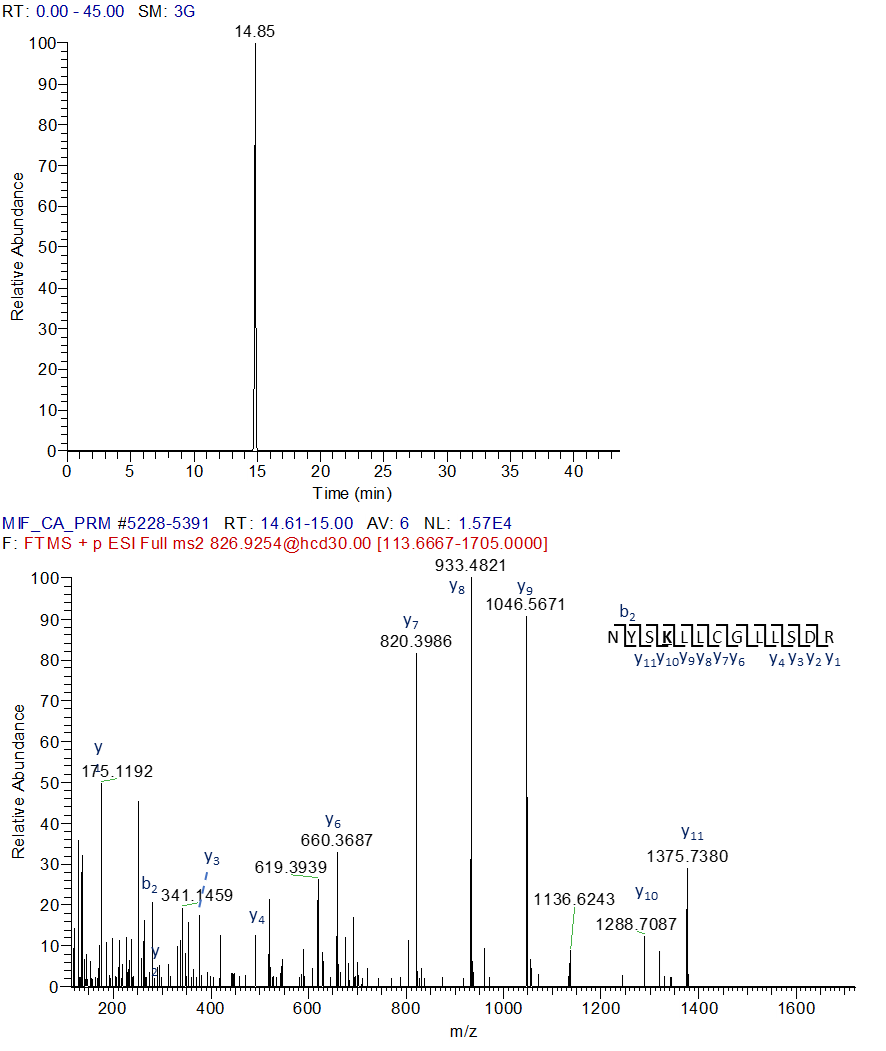


**Figure S15**: Characteristic total ion chromatogram (TIC) in (A) and tandem mass spectra in (B), of the adducted MIF peptide NYSKLLCGLLSDR in position Lys77 after incubation with CA, identified using PRM analysis. B-and y-ions are annotated in the MS^2^ spectrum.


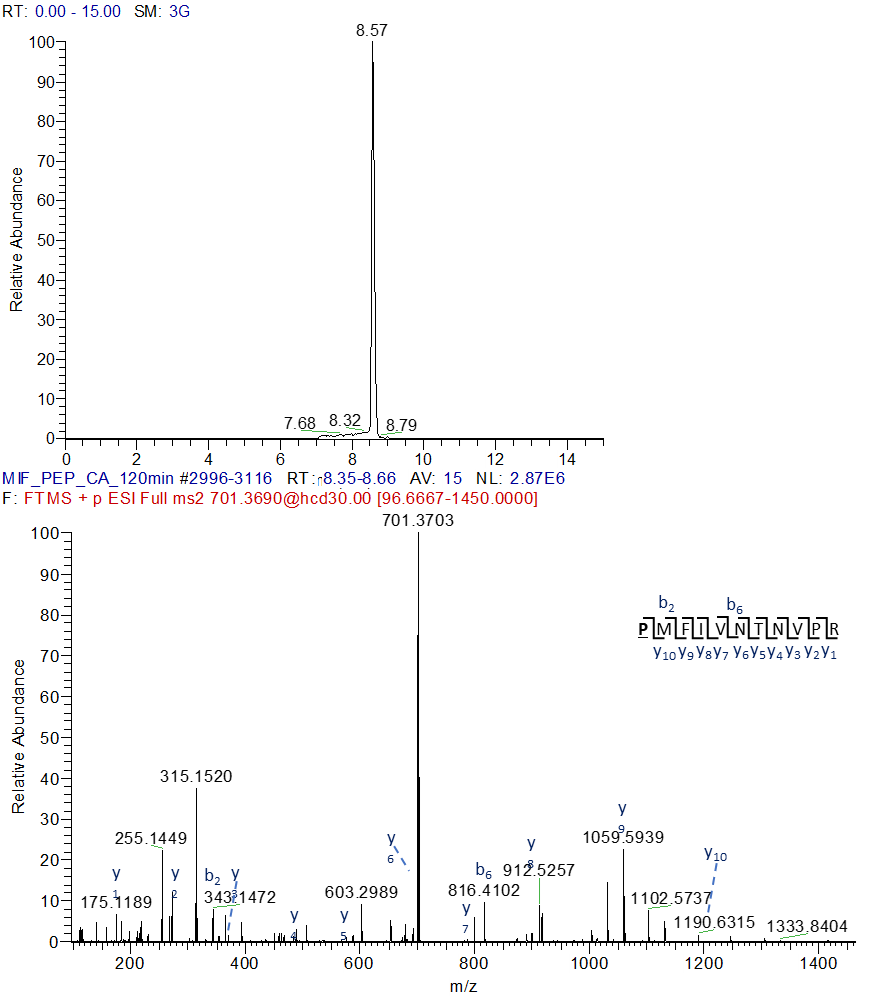


**Figure S16**: Characteristic total ion chromatogram (TIC) in (A) and tandem mass spectra in (B), of the adducted MIF peptide PMFIVNTNVPR in position Pro1 after incubation with CA, identified using PRM analysis. B-and y-ions are annotated in the MS^2^ spectrum.
